# MicroRNA and mRNA expression associated with ectopic germinal centers in thymus of myasthenia gravis

**DOI:** 10.1371/journal.pone.0205464

**Published:** 2018-10-11

**Authors:** Manjistha Sengupta, Bi-Dar Wang, Norman H. Lee, Alexander Marx, Linda L. Kusner, Henry J. Kaminski

**Affiliations:** 1 Department of Neurology, George Washington University, Washington, D.C., United States of America; 2 Department of Pharmacology and Physiology, George Washington University, Washington, D.C., United States of America; 3 Department of Pharmaceutical Sciences, University of Maryland Eastern Shore, Princess Anne, Maryland, United States of America; 4 University Medical Centre Mannheim, University of Heidelberg, Mannheim, Germany; University of Sydney, AUSTRALIA

## Abstract

**Background:**

A characteristic pathology of early onset myasthenia gravis is thymic hyperplasia with ectopic germinal centers (GC). However, the mechanisms that trigger and maintain thymic hyperplasia are poorly characterized. Dysregulation of small, non-coding microRNAs (miRNAs) and their target genes has been identified in the pathology of several autoimmune diseases. We assessed the miRNA and mRNA profiles of the MG thymus and have investigated their role in GC formation and maintenance.

**Methods:**

MG thymus samples were assessed by histology and grouped based upon the appearance of GC; GC positive and GC negative. A systems biology approach was used to study the differences between the groups. Our study included miRNA and mRNA profiling, quantitative real-time PCR validation, miRNA target identification, pathway analysis, miRNA-mRNA reciprocal expression pairing and interaction.

**Results:**

Thirty-eight mature miRNAs and forty-six annotated mRNA transcripts were differentially expressed between the two groups (>1.5 fold change, ANOVA p<0.05). The miRNAs were found to be involved in immune response pathways and identified in other autoimmune diseases. The cellular and molecular functions of the mRNAs showed involvement in cell death and cell survival, cellular proliferation, cytokine signaling and extra-cellular matrix reorganization. Eleven miRNA and mRNA pairs were reciprocally regulated. The Regulator of G protein Signalling 13 (*RGS13*), known to be involved in GC regulation, was identified in specimens with GC and was paired with downregulation of miR-452-5p and miR-139-5p. MiRNA target sites were validated by dual luciferase assay. Transfection of miRNA mimics led to down regulation of *RGS13* expression in Raji cells.

**Conclusion:**

Our study indicates a distinct miRNA and mRNA expression pattern in ectopic GC in MG thymus. These miRNAs and mRNAs are involved in regulatory pathways common to inflammation and immune response, cell cycle regulation and anti-apoptotic pathways suggesting their involvement in support of GC formation in the thymus. We demonstrate for the first time that miR-139-5p and miR-452-5p negatively regulate *RGS13* expression.

## Introduction

Myasthenia gravis (MG) is an autoimmune neuromuscular disorder mediated by antibodies against neuromuscular junction proteins, primarily the acetylcholine receptor (AChR) [1]. A large proportion of AChR antibody positive early onset myasthenia gravis (EOMG) patients have thymic lymphofollicular hyperplasia with ectopic germinal centers (GC) [2] that are rarely observed in thymus of normal individuals. The hyperplastic thymus possesses all the components of the MG immune response with expression of the antigenic target, the AChR or AChR-like proteins [3–5], B cells producing AChR antibodies [6], and AChR autoreactive T cells [7]. The GCs are sites where B cells proliferate, differentiate, undergo selection, and antibody genes undergo somatic hypermutation and class switch. Removal of the thymus improves the clinical course of EOMG patients [8]. These observations make the thymus a likely site of disease initiation and maintenance in MG.

MicroRNAs (miRNAs) are a group of evolutionarily conserved, endogenous, small non-coding RNAs of about 22 nucleotides in length and regulate gene expression post transcriptionally by silencing multiple target genes. MiRNAs bind to complementary 3’ untranslated region (UTR) of target genes causing translational inhibition or degradation of mRNA [9]. In the immune system, miRNAs have been identified as key players in cell development and function [10] and regulation of central and peripheral tolerance [11]. Aberrant miRNA expression has been found in several autoimmune diseases including human studies and animal models of multiple sclerosis, rheumatoid arthritis, and systemic lupus erythematosus [12–16].

Studies have identified alteration of miRNA expression in peripheral blood mononuclear cells (PBMC), sera and thymus from MG patients. Expression profiles of peripheral blood mononuclear cells (PBMC) identified dysregulated miRNAs in MG patients [17–20]. More specifically, Zhang *et al* have demonstrated the association of miR-181c expression to pro-inflammatory cytokines in the PBMCs from MG patients [21]. As well, the miR-150-5p, which influences T cell differentiation [22] has been a particular focus of evaluation, being found to be increased in MG patient sera with its reduction correlating with clinical improvement after thymectomy [23]. MiR-21-5p, which influences T cell responsiveness, has also been found elevated in circulation, while miR-27a-3p, a modulator of NK cells, is significantly reduced in MG serum [23]. Studies on the thymus from MG patients suggest a down regulation of miR-7-5p would alter the expression level of CCL21 resulting in germinal center formation [24]. As well, Li et al have found the reciprocal expression of miR-548k with CXCL13 [25]. These studies suggest that the altered miRNA signatures from MG patients have pathological effects.

Despite decades of investigation of the thymic pathogenesis of MG, the molecular mechanisms, which support the pathological T cell and autoantibody generation, are poorly understood. We evaluated a subset of thymus tissue collected during a randomized controlled study of thymectomy [8] and assessed miRNA and mRNA expression patterns based on the presence or absence of germinal centers in tissue blocks [26]. We demonstrate 1) miRNA and transcriptional profiles of MG thymus segregate based on the presence of germinal centers, 2) differently regulated transcripts fall into categories related to immune response, cell proliferation and cell communication/movement, 3) signaling pathways that are differentially identified include cell death and survival as well as inflammation, while 4) differentially regulated miRNAs are primarily involved in immune response and cell proliferation/apoptosis, and 5) each of the profiles are consistent with other investigations which have identified miRNA and gene transcripts involved in GC formation and maintenance in non-thymic, secondary lymphatic structures.

## Materials and methods

### Samples and preparation

Thymic specimens were obtained over the course of the MGTX study (NS 42685) [8]. The trial evaluated the efficacy of extended transsternal thymectomy in combination with a standardized prednisone protocol. Thymectomized patients showed an improvement in clinical outcomes over a 3-year period, compared to prednisone alone non-thymomatous MG patients. The NINDS funded the trial and assembled a Data Safety Monitoring Board (DSMB) to independently monitor study activities. Sites received local institutional review board/ethics committee approvals (S1 Appendix), and each patient provided written informed consent before study entry including provision of thymic specimens. All specimens were deidentified. The George Washington University institutional review board provided additional review and approved these investigations.

Subjects were between 18 and 65 years old with generalized MG for less than 5 years with elevated serum AChR antibody levels participated in the MGTX study. Cryopreserved thymus in OCT was collected over the course of the MGTX trial according to a specific protocol and 6 blocks were collected from specific region of the tissue [8, 26]. Thymus blocks were used from 17 subjects for microarray analysis (Table 1). From a single tissue block for each subject, 10 micron cryosections underwent staining with hematoxylin and eosin and then assessed for presence of germinal centers. Images were captured using a Leica DM 600-B microscope (Leica Microsystems Inc., Buffalo Grove, IL). Two independent observers assessed sections for presence (GC positive) or absence of germinal centers (GC negative). Remaining tissue was transferred to RNAlater ICE (Ambion, Life Technologies, Carlsbad, CA) frozen tissue transition solution and stored at -20°C for subsequent RNA isolation.

**Table 1:** Clinical information of the subjects and GC score of the tissue blocks used in the study.

Age	Gender	Prednisone dose (mg) at baseline	Prednisone frequency	GC Score (miRNA array)	GC Score (mRNA array)
41	F	40	alternate day	Positive	Positive
41	M	None	N/A	Negative	Positive
22	F	25	daily	Positive	Positive
46	F	None	N/A	Negative	Positive
54	F	None	N/A	Negative	Not Used
57	F	20	alternate day	Negative	Not Used
21	F	75	alternate day	Positive	Negative
34	F	30	daily	Positive	Negative
63	F	30	daily	Negative	Not Used
24	M	50	daily	Positive	Positive
29	F	20	alternate day	Not Used	Negative
59	F	100	alternate day	Negative	Negative
56	M	100	alternate day	Negative	Not Used
22	F	10	daily	Negative	Negative
37	F	None	N/A	Positive	Positive
18	F	35	daily	Negative	Negative
19	F	10	daily	Positive	Positive

Due to heterogeneity of thymus tissue, individual tissue blocks were inspected for the presence of GCs and grouped accordingly. N/A not applicable.

### RNA extraction and sample use

For mRNA profiling, thymus samples were placed in Trizol (Ambion, Life Technologies, Carlsbad, CA) to extract total RNA. For the miRNA analysis, total RNA was extracted from thymus blocks using mirVana miRNA isolation kit (Ambion) according to the manufacturer’s protocol. The crude RNA samples were treated with RNase Free DNase (QIAGEN, Valencia, CA) and were purified using RNeasy mini kit (QIAGEN). For miRNA samples, we followed manufacturer recommended protocol for small RNA purification. RNA was quantitated using a NanoVue plus spectrophotometer (GE Healthcare Lifesciences, Marlborough, MA). The purity was assessed by optical density ratio of OD260/OD280 and was required to be greater than 1.8. RNA integrity was assessed by gel electrophoresis and using the Agilent 2100 Bioanalyzer (Agilent Technologies, Santa Clara, CA). Samples with RIN value greater than 7.0 were used for expression profiling. For miRNA assessment, sixteen samples (7 GC positive and 9 GC negative) and for RNA profiling 13 samples (7 GC positive and 6 GC negative) met criteria for use in microarray studies.

### Microarray experiments

For miRNA array analysis, 250ng of total RNA was labeled by FlashTag Biotin HSR RNA Labeling Kit (Affymetrix, Inc., Santa Clara, CA). Target labeling was confirmed by ELOSA QC assay as suggested by Affymetrix. Biotin labeled samples with eukaryotic hybridization control were hybridized to GeneChip miRNA 4.0 Array (Affymetrix) chips in a Gene Chip hybridization Oven 640 (Affymetrix) for 18hrs. The Gene Chip Fluid Station 450 was used for washing and staining of the arrays. These were then scanned by an Affymetrix GeneChip Scanner (GC30007G). All procedures were done as recommended by the manufacturer. Affymetrix CEL files were processed and quantile normalized with GC-RMA background correction using the Expression Console and Transcriptome Analysis Console (TAC) 2.0 (Affymetrix). Differential expression of miRNAs was statistically evaluated using two independent programs: Partek Genomic Suite 6.6 (Partek, St. Louis, MO) and TAC 2.0. ANOVA p-value less than 0.05 was considered as significant. MiRNAs with greater than 1.5 fold difference in abundance between GC positive and GC negative samples as identified by both the programs were selected for further validation by quantitative Real-time PCR (qRT-PCR). This approach yielded 96% qRT-PCR validation success (see Results section). Ingenuity Pathway Analysis (IPA) (QIAGEN) miRNA Target filter was used to identify potential targets of miRNA. Experimentally observed or highly predicted confidence levels as specified by the program were used for target determination. Ingenuity Pathway Analysis (IPA) was used to predict pathways and networks.

For mRNA array analysis, GeneChip WT plus reagent kit (Affymetrix) was used for single stranded cDNA synthesis and labeling of 300ng of total RNA. The samples were hybridized to GeneChip human transcriptome array (HTA) 2.0 (Affymetrix). The chips were scanned by GeneChip Scanner (Affymetrix) and CEL files were generated. Expression console was used to generate CHP files from the CEL files. The array data were normalized by quantile normalization with GC-RMA background correction; data visualization and statistical analysis were performed as described for miRNA expression analysis with a qRT-PCR validation success rate of 100% (see Results section). Gene ontology (GO) enrichment analysis was performed for annotated genes with greater than 1.5 fold difference in expression level. The gene sets were mapped to GO terms according to biological process and molecular functions. Bonferroni correction for multiple testing was used. IPA core analysis was performed to identify molecular and cellular functions, canonical pathways and networks.

Microarray data has been submitted to GEO and can be viewed by accession numbers GSE103812 and GSE103974 for GeneChip miRNA 4.0 Array and GeneChip human transcriptome array 2.0 respectively.

### Statistical analysis

One-way analysis of variance (ANOVA) analysis was performed, comparing GC positive to GC negative samples with the null hypothesis that the mean values of the different miRNAs and mRNA were the same across the categories. Principal component analysis (PCA) plots and hierarchical clustering of mRNA and miRNA data were performed using the Partek Genomics Suite 6.6. Unpaired student’s t-test was performed for qRT-PCR validation of the two groups. P < 0.05 was considered significant.

### Quantitative Real Time-PCR validations

Microarray results were verified by qRT-PCR. For miRNA validation, total RNA (250ng) from fifteen samples with appropriate RNA integrity was reverse transcribed from using NCode VILO miRNA cDNA Synthesis Kit (Invitrogen, Carlsbad, CA) following manufacturer’s protocol. Quantitative RT-PCR was performed from using a Bio-Rad CFX384 Real-Time system (Bio-Rad, Hercules, CA) and SYBR Green PCR Master Mix (Applied Biosystems, Life Technologies, Grand Island, NY). 250nM of universal reverse primer and miRNA specific primers (S1A Table) were used in 14μl PCR reaction mix. The PCR protocol used was 95°C for 10 min, followed by 40 cycles at 95°C for 15 s and 60°C for 1 min. Melt curve analysis was performed at the end as follows: 60°C to 95°C with 0.5°C increment for 10s. The expression level U6 RNA was used as an endogenous control. Comparative threshold cycle (2^(−ΔΔCT)^) method of relative quantitation was used to analyze the expression levels [27].

For mRNA validation studies, 1 μg of total RNA was reverse transcribed from thirteen samples using the Revert Aid First strand cDNA Synthesis Kit (Thermo Scientific, Life Technologies, Grand Island, NY). Real-time PCR was performed using SYBR Green PCR Master Mix and gene specific primers (S1B Table). Data were normalized to levels of the house keeping genes *EIF1AX* for thymic studies and *GAPDH* for cell culture experiments. The 2^(−ΔΔCT)^ method was used to determine expression differences. EIF1AX is constitutively expressed and its expression did not change in both microarray and qRT-PCR experiments, as has been demonstrated in diverse gene expression studies [28–30]. Experiments were done in duplicate. Student’s t-test was used for statistical analysis. P<0.05 was considered as significant.

### Cell culture and transfection

Raji and 293T cell lines were obtained from ATCC (Manassas, VA). 293T cell line was grown in DMEM (Gibco, Thermo Fisher Scientific, Grand Island, NY) medium with 10% heat inactivated fetal bovine serum (FBS) (Gibco) and 1% penicillin-streptomycin (Gibco). Raji cell line was grown in RPMI 1640 (Gibco) with 10% fetal bovine serum (FBS) (Gibco) and 1% penicillin-streptomycin (Gibco). The cells were cultured at 37°C with 5% CO_2_.

Raji cells were transfected with miRNA mimic using reverse transfection protocol. Transfection was performed in 6 well plates. 50nM/L of *mir*Vana miRNA mimics (Ambion) hsa-miR-139-5p, hsa-miR-452-5p and Cy3 Dye-Labeled Pre-miR Negative Control #1 (Invitrogen) were suspended in 500μl of Opti-MEM I low-serum medium (Gibco). The RNA mixture was added to each well, followed by five μl of Lipofectamine RNAiMAX (Invitrogen, Life Technologies, Carlsbad, CA). Plates were incubated for 10–20 minutes at room temperature. Raji cells (2.5 x 10^5^) in RPMI 1640 medium containing 10% FBS without antibiotics were added to each well. Cells without RNA served as controls. Transfection was done in triplicates. Cells were incubated in 5% CO_2_ at 37°C and collected 72 hours post transfection for RNA isolation by Trizol reagent (Ambion). Transfection efficiency was 80% and was calculated by percentage of fluorescent cells in the negative control wells. One microgram of total RNA was used for cDNA synthesis and qRT-PCR was performed for determining *RGS13* expression. Average of three independent experiments was plotted. Student’s t-test was performed and p<0.05 was taken as significant.

### Dual luciferase assay

The predicted target sites of miR-139-5p and miR-452-5p present in 3’UTR of *RGS13* were cloned into SacI and XbaI sites of pmirGLO Dual-Luciferase miRNA Target Expression Vector (Promega, Madison, WI). Synthetic oligonucleotides (Eurofins Genomics, Louisville, KY) corresponding to 70 nucleotides surrounding the target sequences were annealed and ligated to pmirGlo plasmid. The sequences are provided below. The underlined sequences define the miRNA binding sites as identified by TargetScan:

RGS13_139_Sense
  5’CGCGGCCGCAGGTCTTTCTTCATGATACAAGCATTATAAAGTTTTTACTGTAGTAGTCAATTAATGGATT 3’
RGS13_139_Antisense
  5’CTAGAATCCATTAATTGACTACTACAGTAAAAACTTTATAATGCTTGTATCATGAAGAAAGACCTGCGGCCGCGAGCT 3’
RGS13_452_Sense
  5’CGCGGCCGCCTACAACTCAAAAGTTTAAATAGAAAACAGTATATTGAAAGTGGTGGGTTTGATCTTTTTT 3’
RGS13_452_Antisense
  5’CTAGAAAAAAGATCAAACCCACCACTTTCAATATACTGTTTTCTATTTAAACTTTTGAGTTGTAGGCGGCCGCGAGCT 3’

All the constructs were verified by sequencing. The 293T cell line was used for transfection. 6 x 10^4^ cells were plated in each well in a 48 well plate. The empty vectors with cloned seed sequences did not produce any change in luciferase activity when compared to parent pmirGLO. A dose response of RNA determined the optimal concentration at 10nM. The cells were transfected with 50ng of plasmid constructs and 10nM miRNA mimic or negative control mimic (Ambion) by Lipofectamine 2000 (Thermo Fisher Scientific). Luciferase activity was assayed 48 hours post transfection using Dual-Luciferase Reporter Assay System (Promega) and detected on Glo-Max Multi Jr luminometer (Promega). Firefly luciferase activity was normalized to the Renilla luciferase activity as internal control in each well. Assays were conducted in triplicate and three independent experiments were performed. The data was plotted as mean and SEM of three independent experiments. Student’s t-test was performed and p<0.05 was considered as significant.

## Results

This study used thymus blocks from the MGTX trial [8], which showed benefit of thymectomy. Inclusion criteria allowed for the use of glucocorticoids prior to thymectomy. Of the seventeen thymuses, 16 blocks were assessed for miRNA and 13 blocks were designated for mRNA (Table 1). Due to the heterogeneity of the thymus, each block was assessed and categorized based on the presence or absence of GC (S1 Fig). By structuring the analysis into the two groups, the results allow for the identification of the most critical expression differences that drive thymic changes to formation and maintenance of GCs.

### Characteristics of miRNA expression in MG thymus

**Fig 1:**
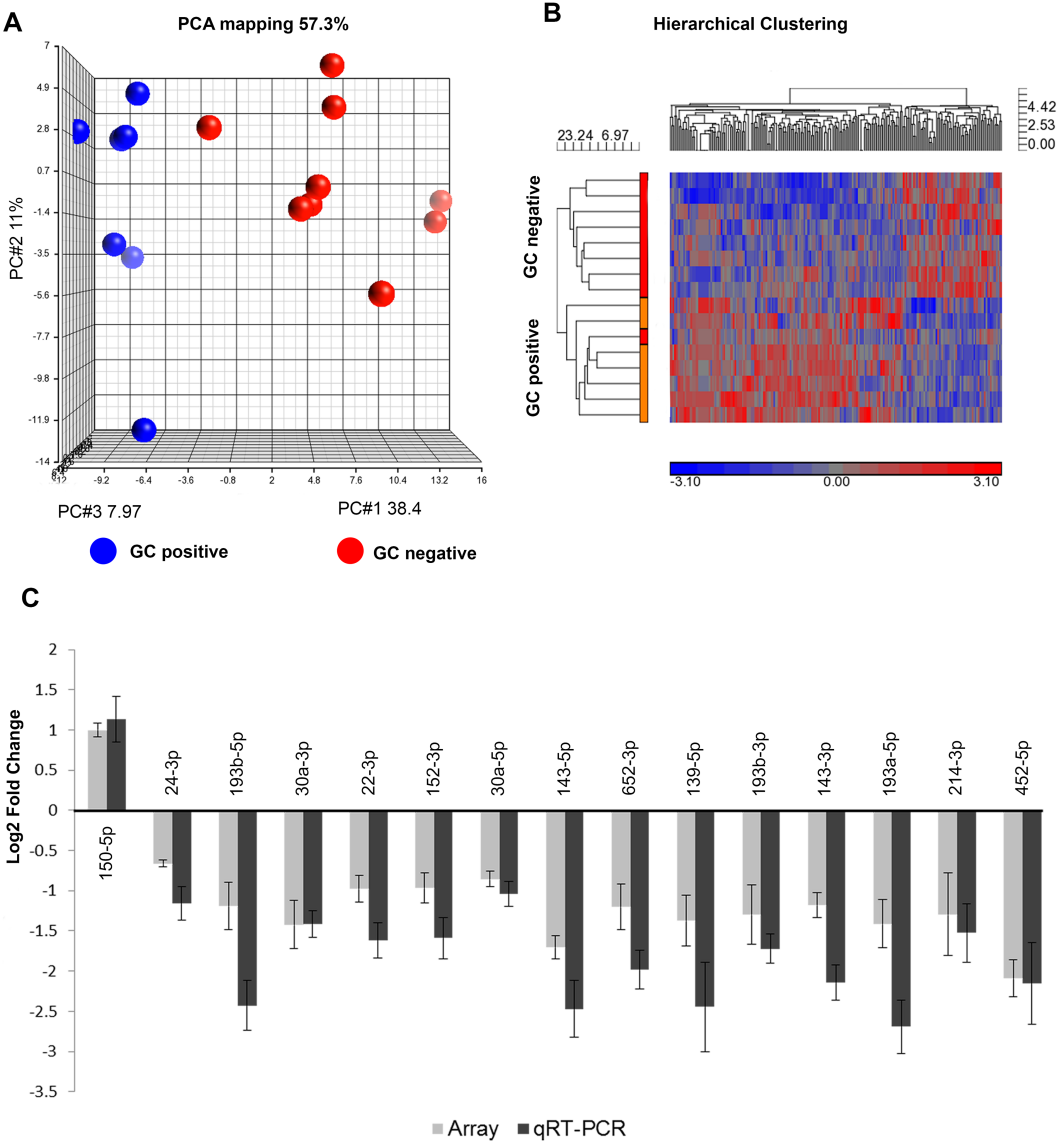
Differential small RNA expression profiles of MG thymus samples with and without germinal centers (GC). (A) Principal Component Analysis (PCA) plot. (B) Hierarchical clustergram plot (ANOVA p<0.05, no fold change). The plots demonstrate clear separation between the groups. GC positive n = 7; GC negative n = 9. (C) qRT-PCR validation of miRNA expression array results. The gray bars represent array data while the black bars represent qRT-PCR data. Log2 fold change has been plotted. Error bars represents +/- SEM. The qRT-PCR results that showed significant differences (as determined by student’s t test, p<0.05) between the GC positive vs GC negative groups have been plotted.

Principal component analysis and two-dimensional hierarchical cluster analysis distinguished miRNA profiles of GC positive and negative thymic specimens (Fig 1A and 1B). A total of 55 non-coding RNAs showed greater than 1.5-fold difference in expression between the groups (S2A Table). Of those, 38 mature miRNAs were identified (S2B Table). Based on the differential expression levels of the matured miRNAs as determined by Partek genomic suite and TAC, we selected 24 miRNAs for verification by qRT-PCR and of those, 23 matched array results (S2B Table). Fifteen miRNAs showed statistically significant differences between the two groups as determined by student’s t-test (Fig 1C, S2 Fig).

IPA miRNA target filter using TargetScan identified targets for 28 miRNAs out of 38 mature miRNAs with confidence level high or previously experimentally identified. Canonical pathways identified were related to humoral immune response, cellular immunity, cytokine, and NF kappa B signaling (S3A Table). In addition, cell cycle regulation, cellular growth, proliferation, cancer and apoptotic pathways were recognized (S3B Table). Considerable overlap with miRNAs previously identified to be involved in systemic lupus erythematosus, rheumatoid arthritis and autoimmune thyroid were identified (S3C Table). Only, two miRNAs in the analysis had been associated with multiple sclerosis.

**Fig 2:**
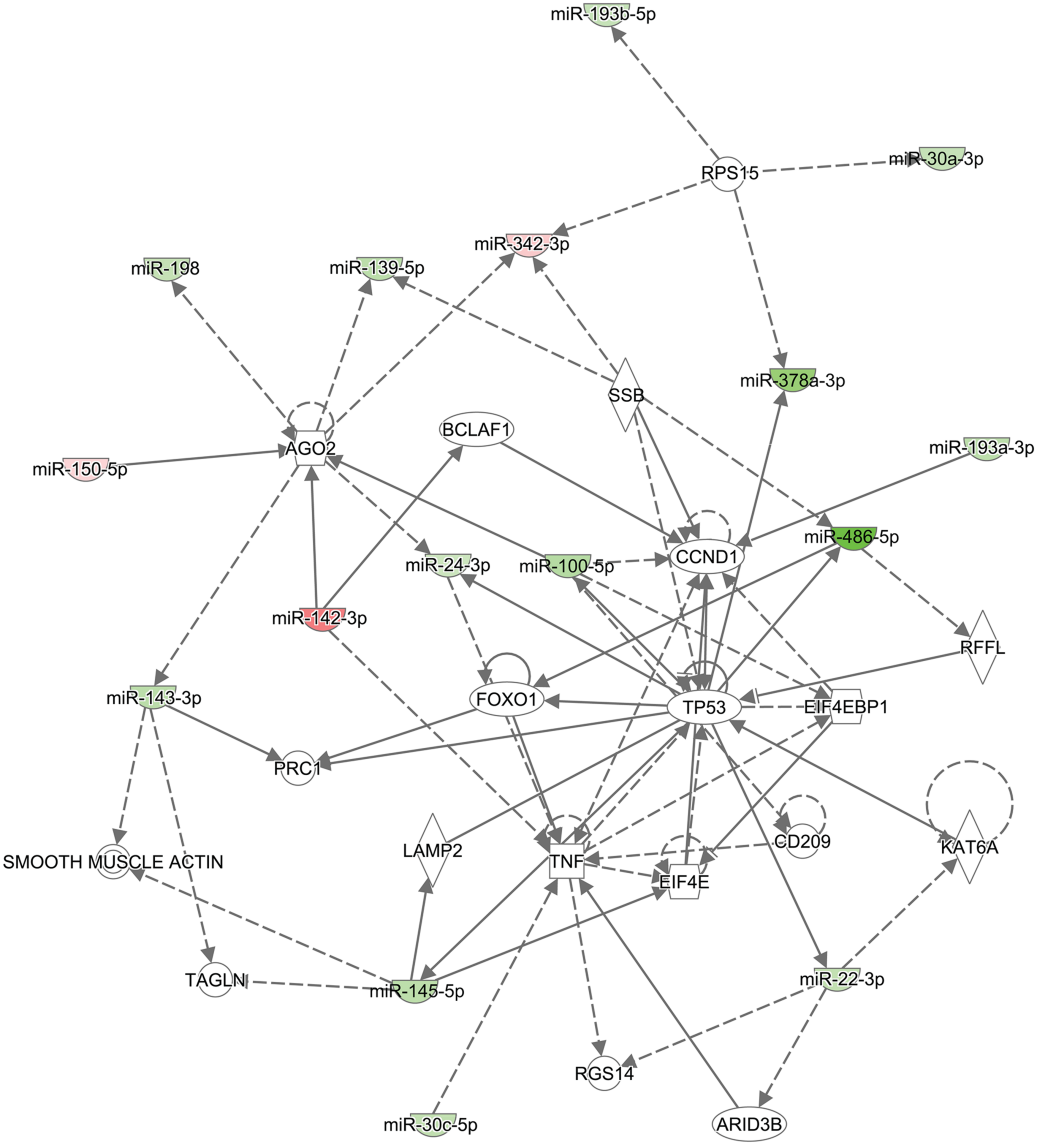
IPA core analysis program identified micro RNA network involved in cancer, gastrointestinal disease, organismal injury and abnormalities pathways. The solid arrows indicate direct interactions and the dotted arrows indicate indirect interaction between the differentially expressed miRNA and their predicted targets. The miRNAs that are over expressed in GC positive samples are marked in red and those repressed are marked in green.

IPA core analysis revealed diseases and biological functions known to be regulated by the 38 selected mature miRNAs. Twenty-four miRNA were associated with cancers (p <0.048 to 2.13 x 10^−14^). Twelve were associated with hematological disorders (p < 0.02 to 2.69 x 10^−13^) and ten with immunological disorders (p <0.004 to 2.69 x 10^−13^). Molecular and cellular functions affected included cellular growth and proliferation (10 miRNAs; p<0.02 to 1.60 x 10^−5^), cellular development (11 miRNAs; p<0.02 to 1.60 x 10^−5^) and cell death-cell survival (11 miRNAs; p< 0.02 to 0.0004). The most common networks in which the differentially expressed miRNAs participate were cancer, gastrointestinal disease, organismal injury and abnormalities (Fig 2) with 16 miRNA predicted to be involved in these networks. IPA core analysis identified a broad range of regulatory networks because the select miRNAs have multiple potential targets.

### Gene expression analysis

**Fig 3:**
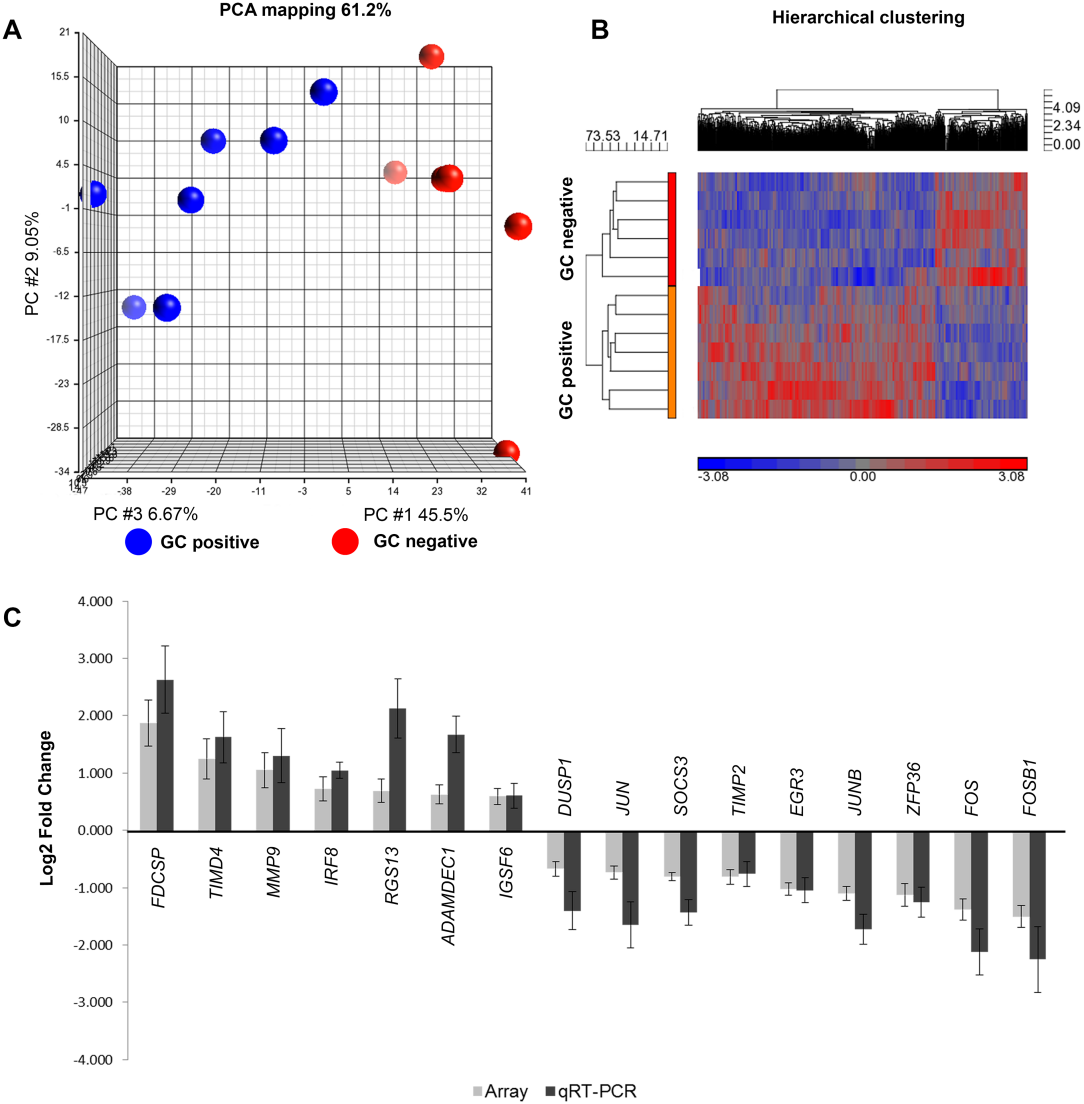
Differential mRNA expression profiles of thymus samples with and without germinal centers. (A) PCA plot and (B) Hierarchical clustergram plot (ANOVA p<0.05, no fold change). GC positive n = 7; GC negative n = 6. The plots demonstrate clear separation between the groups. (C) qRT-PCR validation of mRNA expression array results. The gray bars represent array data while the black bars represent qRT-PCR data. Log2 fold change has been plotted. Error bars represents +/- SEM. The qRT-PCR results that showed significant differences (as determined by student’s t test, p<0.05) between the GC positive vs GC negative groups have been plotted.

Transcriptional profiling identified 2,172 genes to be differentially expressed between the two groups (ANOVA p<0.05). Fig 3 shows the clear separation of groups based on PCA (Fig 3A) and hierarchical cluster analysis (Fig 3B). Applying our stringent selection criterion, 99 transcripts had a greater than 1.5 fold differential expression. Of the 99 total transcripts identified, 46 transcripts are annotated (Table 2). Based on a range of expression differences of array results, 24 transcripts were selected for verification by qRT-PCR and a 100 percent concordance was found in the direction of expression difference (S4 Table). Sixteen of the gene transcripts demonstrated statistically significant differences between GC-positive and GC-negative groups (Fig 3C, S3 Fig).

**Table 2:** Differentially expressed annotated transcripts.

Gene Symbol	RefSeq	Gene assignment	Fold Change	ANOVA p-value
*FDCSP*	NM_152997	follicular dendritic cell secreted protein	3.661	0.015
*CCL21*	NM_002989	chemokine (C-C motif) ligand 21	3.604	0.036
*TIMD4*	NM_001146726	T-cell immunoglobulin and mucin domain containing 4	2.376	0.023
*AC079767*.*4*	OTTHUMT00000337055	novel transcript	2.192	0.014
*MMP9*	NM_004994	matrix metallopeptidase 9	2.071	0.024
*SNORD9*	NR_003029	small nucleolar RNA	1.948	0.021
*RNASE6*	NM_005615	RNASE6	1.835	0.034
*SNORD90*	NR_003071	small nucleolar RNA	1.827	0.023
*HLA-DRB1*	NM_001243965	major histocompatibility complex, class II, DR beta 1	1.779	0.026
*NAPSB*	NR_002798	napsin B aspartic peptidase, pseudogene	1.744	0.022
*CTSH*	Y18461	cathepsin H	1.740	0.044
*IRF8*	NM_002163	interferon regulatory factor 8	1.655	0.037
*RGS13*	NM_002927	regulator of G-protein signaling 13	1.613	0.027
*VCAM1*	NM_001078	vascular cell adhesion molecule 1	1.611	0.016
*RNY5*	ENST00000516501	RNA, Ro-associated Y5	1.609	0.048
*SCARNA5*	AY077737	small Cajal body-specific RNA 5	1.590	0.034
*SCARNA5*	NR_003008	small Cajal body-specific RNA 5	1.584	0.028
*ADAMDEC1*	NM_001145271	ADAM-like, decysin 1	1.545	0.021
*TYROBP*	NR_033390	TYRO protein tyrosine kinase binding protein	1.531	0.037
*IGSF6*	NM_005849	immunoglobulin superfamily, member 6	1.507	0.012
*PLA2G7*	NM_001168357	phospholipase A2, group VII	1.506	0.039
*DKFZP586I1420*	NR_002186	uncharacterized protein DKFZp586I1420	1.504	0.048
*PXN*	BC052611	paxillin	-1.544	0.035
*NR4A1*	NM_001202233	nuclear receptor subfamily 4, group A, member 1	-1.584	0.018
*DUSP1*	NM_004417	dual specificity phosphatase 1	-1.593	0.023
*KLF10*	X75691	Kruppel-like factor 10	-1.598	0.036
*ATF3*	NM_001030287	activating transcription factor 3	-1.619	0.015
*GADD45B*	AY615271	growth arrest and DNA-damage-inducible, beta	-1.627	0.008
*JUN*	NM_002228	jun proto-oncogene	-1.661	0.022
*CH25H*	NM_003956	CH25H	-1.692	0.038
*KRT17*	NM_000422	keratin 17	-1.748	0.043
*SOCS3*	NM_003955	suppressor of cytokine signaling 3	-1.751	0.006
*TIMP2*	X54533	TIMP metallopeptidase inhibitor 2	-1.755	0.018
*LOC284454*	NR_036515	uncharacterized LOC284454	-1.799	0.035
*GADD45B*	NM_015675	growth arrest and DNA-damage-inducible, beta	-1.816	0.006
*JUNB*	DQ650707	jun B proto-oncogene	-1.885	0.024
*ADAMTS1*	NM_006988	ADAM metallopeptidase with thrombospondin type 1 motif, 1	-1.914	0.028
*EGR3*	S40832	early growth response 3	-2.030	0.012
*JUNB*	NM_002229	jun B proto-oncogene	-2.136	0.008
*ZFP36*	NM_003407	ZFP36 ring finger protein	-2.182	0.010
*RASD1*	NM_001199989	RAS, dexamethasone-induced 1	-2.225	0.034
*MEG3*	NR_002766	maternally expressed 3 (non-protein coding)	-2.289	0.026
*EGR1*	NM_001964	early growth response 1	-2.439	0.024
*FOS*	NM_005252	FBJ murine osteosarcoma viral oncogene homolog	-2.597	0.012
*FOSB*	NM_001114171	FBJ murine osteosarcoma viral oncogene homolog B	-2.837	0.017
*FOSB*	D16366	FBJ murine osteosarcoma viral oncogene homolog B	-3.874	0.016

Differentially expressed annotated transcripts in GC positive vs GC negative thymus samples with greater than 1.5 fold change in expression level (ANOVA<0.05).

**Fig 4:**
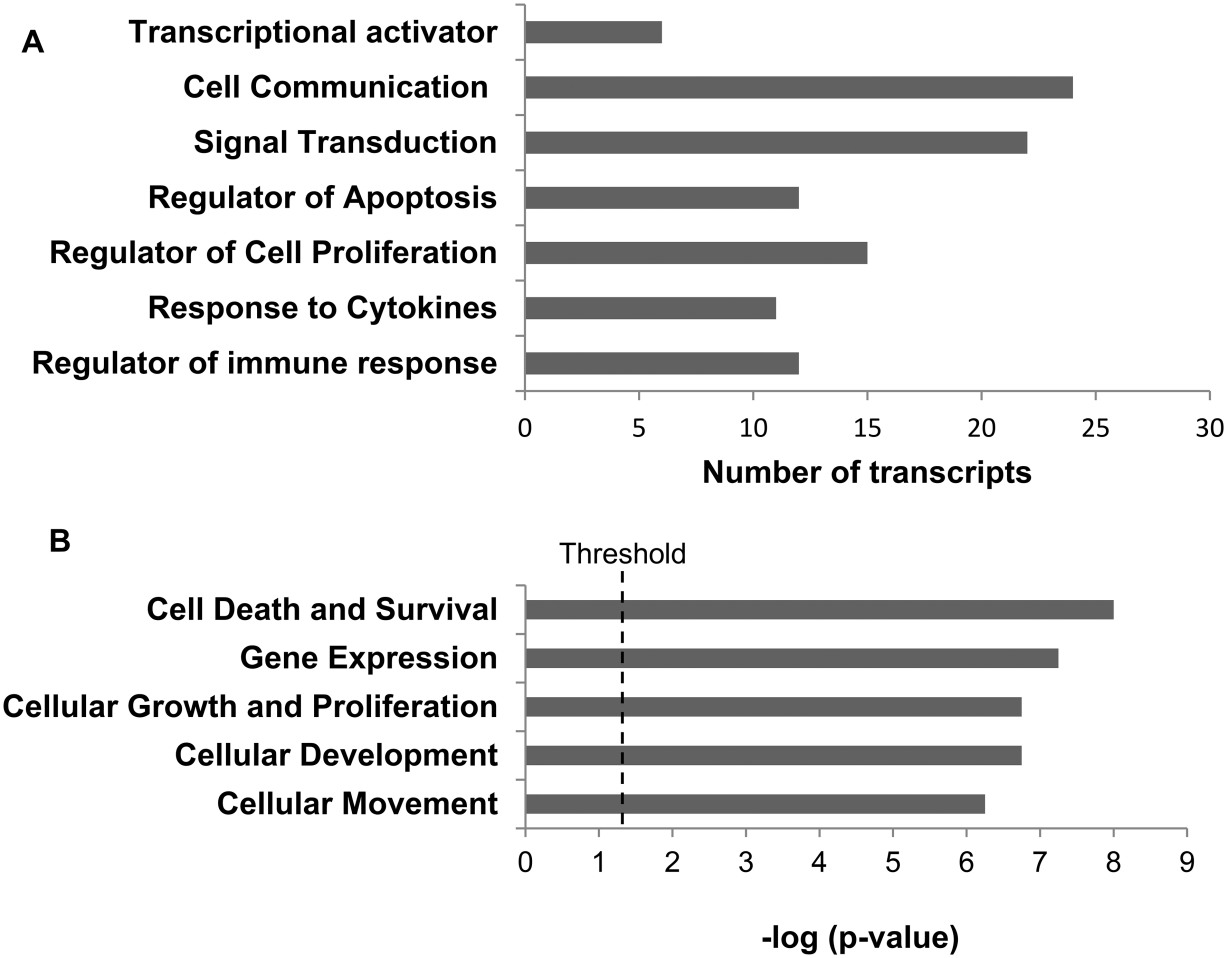
**A) Biological and molecular processes with over representation of transcripts as identified by GO.** The bars represent the number of transcripts in each process. **B) Top five molecular and cellular functions of the differentially expressed genes as identified by IPA analysis.**

Gene ontology enrichment analysis demonstrated over representation of gene expressed in the following major biological processes: regulators of immune response, response to cytokines, cell proliferation, and apoptosis as well as signal transduction, cell communication and transcriptional activation (Fig 4A). Using a 1.5 fold cut-off, IPA identified five major molecular and cellular functions; cell death-cell survival (p< 0.05 to 2.40 x 10^−8^), gene expression (p< 0.05 to 2.86 x 10^−7^), cellular growth and proliferation (p<0.05 to 1.27 x 10^−10^), cellular development (p< 0.05 to 3.57 x 10^−9^) and cell movement (p<0.05 to 5.01 x 10^−9^) (Fig 4B). The enrichment of genes in these categories indicates the importance of cell proliferation functions in GC formation in MG.

**Fig 5:**
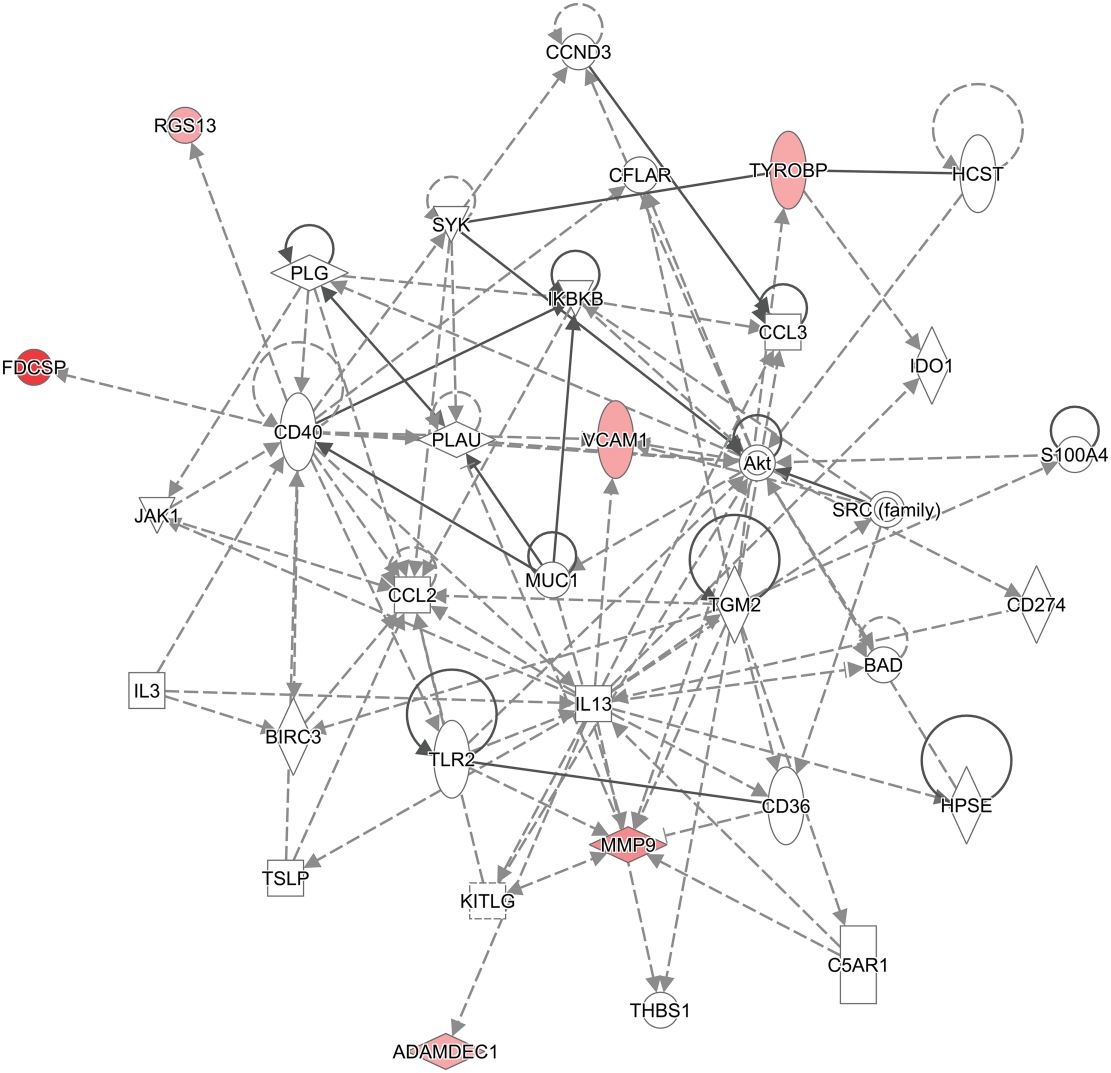
Hematological system development and function, tissue morphology, and inflammatory response network. Gene associated network function was identified by IPA core analysis. The solid arrows indicate direct interactions and the dotted arrows indicate indirect interaction between the differentially expressed mRNA. The over expressed genes in GC positive samples are marked in red.

IPA core analysis identified the major canonical pathways, CXCR4 signaling (p<0.0002) and IL-10 signaling, (p<0.0003) as well as immune cell trafficking (p-value 0.04 to 7.62 x 10^−6^) consistent with expectation that thymus enriched in germinal centers would possess differentially regulated genes involved in the immune response. The gene network identified are involved in neurological disease, organismal injury and abnormalities, hematological system development and function with a score 43 (S4A Fig). The gene network also identified cell death and survival, cellular development, cellular growth and proliferation with scores of 18 (S4B Fig). Hematological system development and function, tissue morphology, and inflammatory response networks (score 10) were identified, which included the Regulator of G protein signaling 13 (*RGS13*) (Fig 5). *RGS13* is known to regulate signaling via G-proteins and is expressed in germinal center B-cells [31]. Taken together, the results indicate that GC enriched thymus has a distinct gene expression signature with genes primarily involved in inflammatory pathways as well as cell proliferation, cell communication and apoptosis.

### Reciprocal pairing analysis

Using the IPA miRNA target filter, 40 miRNA-mRNAs pairs from our differentially expressed transcripts and miRNAs were identified and of these, 11 pairs demonstrated reciprocal expression of miRNA-mRNA with high to moderate confidence level (Table 3).

**Table 3:** MiRNA-mRNA reciprocal expression pairing.

miRNA ID	Fold Change in expression of miRNA	Confidence level	Target Gene Symbol	Fold Change in expression of Target Gene
hsa-miR-142-5p	8.02	Moderate (predicted)	*KLF10*	-1.598
hsa-miR-718	2.48	High (predicted)	*EGR3*	-2.03
hsa-miR-718	2.48	Moderate (predicted)	*SOCS3*	-1.751
hsa-miR-342-3p	2.04	High (predicted)	*FOSB*	-3.874
hsa-miR-3194-3p	1.51	Moderate (predicted)	*ATF3*	-1.619
hsa-miR-145-5p	-2.07	Moderate (predicted)	*VCAM1*	1.611
hsa-miR-139-5p	-2.09	Moderate (predicted)	*RGS13*	1.613
hsa-miR-214-3p	-3.32	Moderate (predicted)	*RNASE6*	1.835
hsa-miR-378d	-3.5	Moderate (predicted)	*ADAMDEC1*	1.545
hsa-miR-452-5p	-3.99	Moderate (predicted)	*IRF8*	1.655
hsa-miR-452-5p	-3.99	Moderate (predicted)	*RGS13*	1.613

Reciprocal pairing of miRNA and mRNA between GC positive vs GC negative groups as determined by IPA analysis; source is Target Scan (Human).

### RGS13 expression and regulation

**Fig 6:**
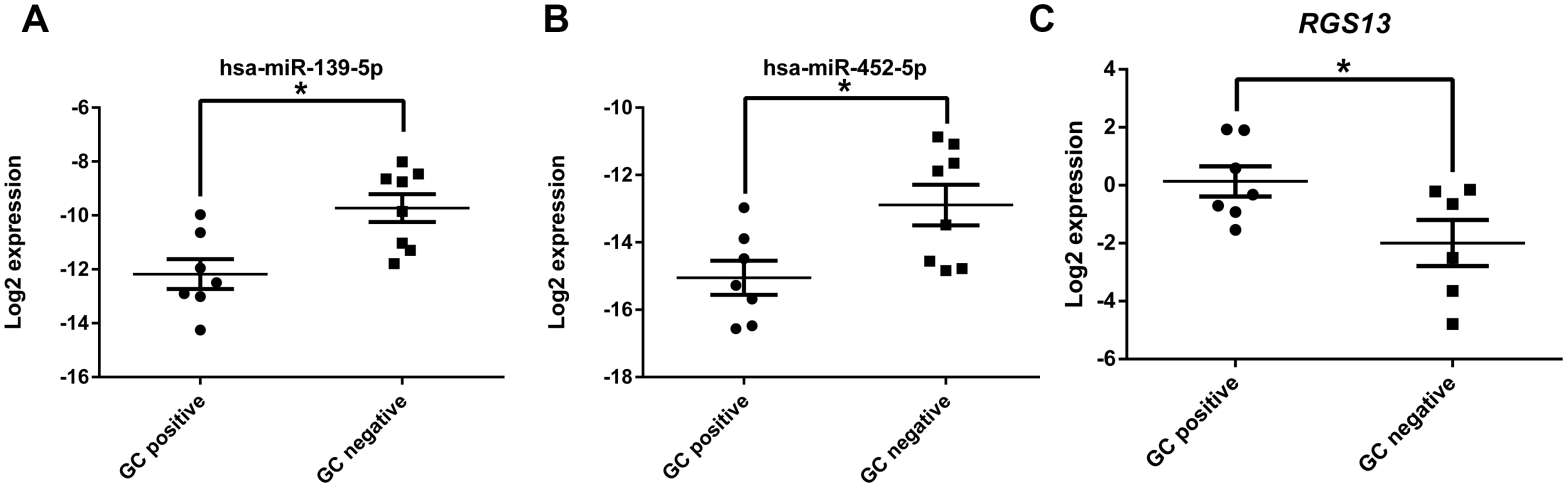
Reciprocal expression of miRNA and mRNA in thymus. qRT-PCR validation of miR-139-5p (A), miR-452-5p (B) and (C) *RGS13* expression in the two groups show reciprocal expression of the micro RNAs and *RGS13* mRNA. Each dot represents data obtained from a patient. It is expressed as +/- SEM. The level of expression for miRNAs was normalized to that of the U6 RNA. *RGS13* expression was normalized to *GAPDH* expression. Student’s t test was performed with p<0.05 considered as significant.

**Fig 7:**
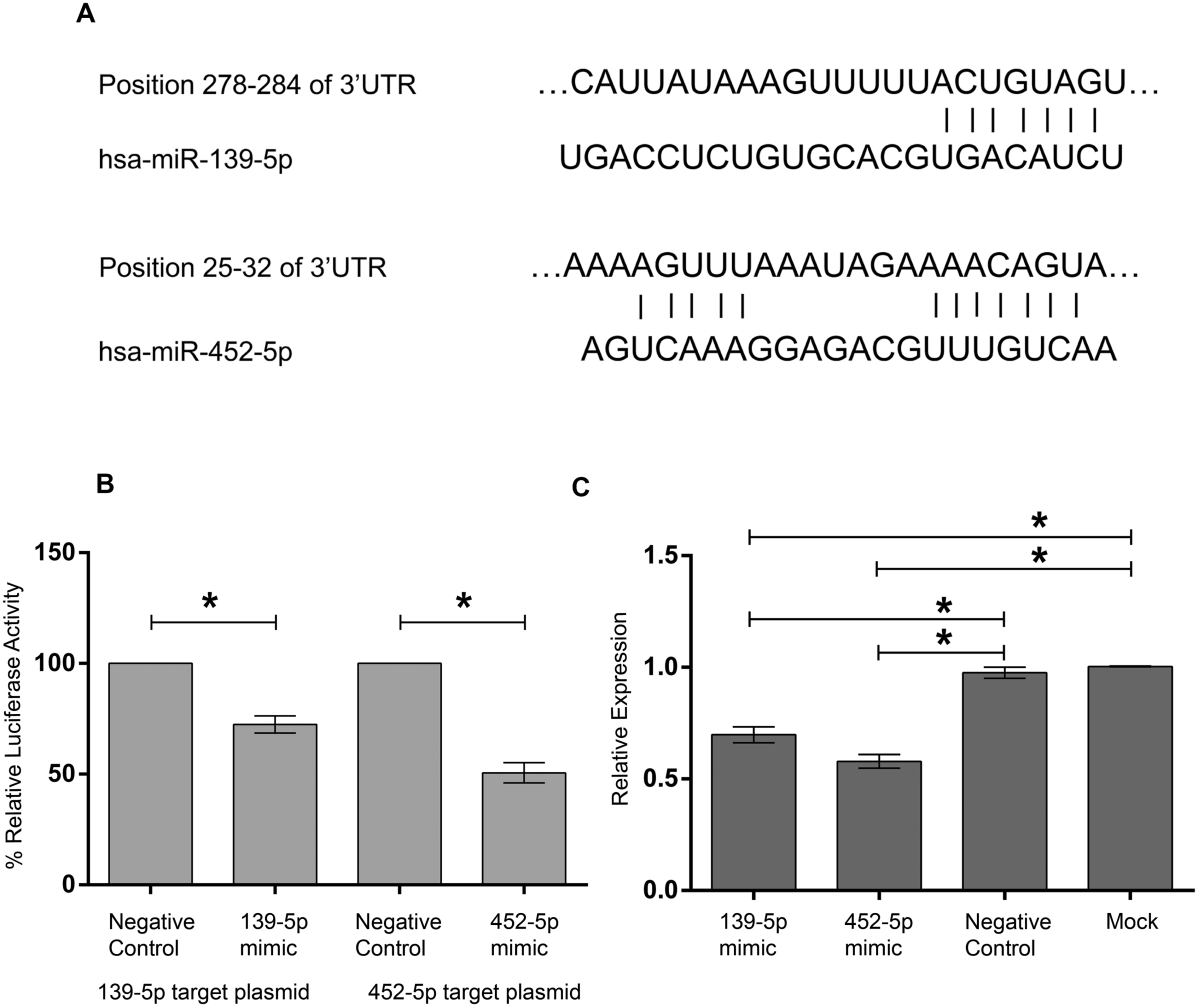
Regulation of RGS*13* expression by miRNAs 139-5p and 452-5p. A) 3’UTR of *RGS13* gene has predicted binding sites for miR-139-5p and miR-452-5p. Sequence alignment between miR-139-5p and miR-452-5p seed sequences with 3’ UTR of human *RGS13* as predicted by TargetScan. Solid line represents seed match region. B) Dual luciferase assay validating miRNA targets in *RGS13* 3’UTR. Predicted target sites of miR-139-5p and 452-5p were cloned into pmirGlo plasmid. 293T cells were co-transfected with target plasmids and 10nM of miRNA mimic or negative control. Luciferase assay was performed after 48 hours post transfection. C) qRT-PCR analysis RGS13 expression in Raji cells post transfection with miRNA mimics of miR-139-5p and miR-452-5p. A nonspecific scramble RNA is used as a negative control. A mock transfection was set up without any RNA. *RGS13* transcript level was reduced after 72 hours of transfection. Human *GAPDH* expression was used as internal control. The data represents average of three independent experiments done in triplicate. The error bars indicates SEM +/-. Student’s t-test was performed, p< 0.05 is considered as significant.

Regulator of G-protein signaling (RGS) family of proteins regulates receptor coupled heterotrimeric G protein by accelerating GTP hydrolysis thereby terminating signaling cascade. RGS13 is expressed GC B cells and thymic epithelial cells. RGS13 along with its homologue RGS1 regulates chemokine responsiveness of GC B cells through chemokine receptors CXCR4 and CXCR5 [31]. As a validation of the reciprocal pairing analysis, we selected *RGS13* and its predicted regulators miR-139-5p and miR-452-5p for further assessment. We verified their expression by real-time PCR and our findings matched our array results. Expression of miR-139-5p and miR-452-5p were significantly lower in GC positive samples (Fig 6A and 6B) whereas *RGS13* expression was higher in GC positive samples (Fig 6C). The seed sequence binding sites for miR-139-5p (5’ CUACAGU 3’) and miR-452-5p (5’ ACUGUUU 3’) were found to have exact matches in the 3’UTR region of *RGS13* (Fig 7A). We cloned about 70 nucleotides surrounding the target sequences of miR-139-5p and miR-452-5p of 3’UTR region of *RGS13* to pmirGLO Dual-Luciferase miRNA Target Expression Vector (pmirGlo). We co-transfected the target vectors with 10nM of miRNA mimic or negative control and assayed for luciferase expression after 48 hours. A significant decrease in luciferase expression was observed with respect to negative control mimic indicating miRNA binding (Fig 7B). We confirmed *RGS13* expression by RT-PCR in Raji cells (S5 Fig) [31, 32], a Burkitt’s lymphoma cell line [33]. After transfection with either miR-139-5p or miR-452-5p mimics, we found a significant reduction in *RGS13* expression at 72 hours post transfection as determined by qRT-PCR (Fig 7C). A scrambled negative control had no effect.

## Discussion

**Fig 8:**
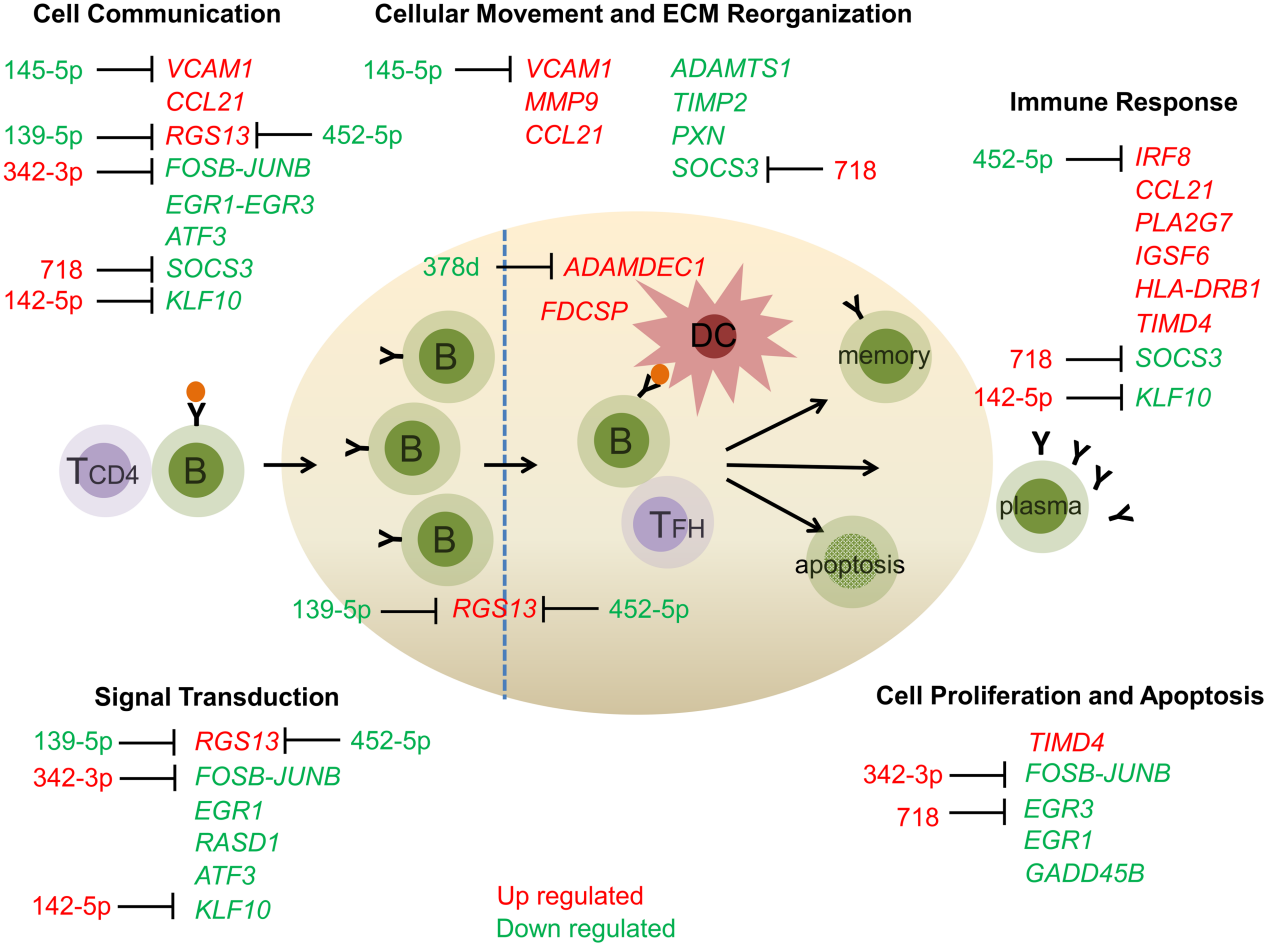
The differentially expressed mRNA in GC positive biopsies highlight molecular and cellular functions that are important for GC formation and maintenance. The differentially expressed transcripts belong to immune response, cellular movement and extracellular matrix reorganization, cell communication, signal transduction and cell proliferation and apoptosis. These pathways are important for GC functions. The transcripts that are upregulated in GC positive samples are marked in red while the ones that are downregulated are marked in green. The miRNAs that are reciprocally expressed with mRNA targets are marked. The transcripts that are known to be expressed by GC B cells and dendritic cells are shown. ECM, Extra cellular matrix.

Our studies of miRNA and gene expression profiles provide an assessment of mechanistic pathways that could underlie germinal center development and maintenance in the MG thymus (Fig 8). We assessed samples from the MGTX trial for the presence of GCs, which allowed the use of glucocorticoids for inclusion into the study. Studies have shown that glucocorticoids reduce the presence of GCs in the thymus and return transcript levels to normal state [34–36]. We demonstrate miRNA and transcriptional profiles of thymus from MG subjects segregate based on the presence of germinal centers. Differently regulated transcripts fall into categories related to immune response, cell proliferation and cell communication/movement, while signaling pathways that are differentially identified by over-representation analysis (i.e. differentially expressed transcripts and miRNAs over-represented in specific signaling pathways) include cell death and survival as well as inflammation. The differentially regulated miRNAs are primarily involved in immune response and cell proliferation/apoptosis. Although IPA placed differentially regulated transcripts into specific categories, a clear functional relationship exists among the differentially expressed transcripts, which identified pathways involved in immune-related response, cell death/survival, and cell migration. Our profiling identified miRNA and gene transcripts known to be involved in germinal center formation and maintenance in secondary lymphatic structures. The differentially expressed miRNAs in MG thymus that we identified have also been linked to systemic lupus erythematosus, rheumatoid arthritis, and autoimmune thyroid disease. The following sections provide a more specific discussion of particular study results.

### Immune-related Pathways

Transcriptional profiling identified a differential representation of transcripts that would support a pro-inflammatory environment within the GC rich MG thymus (*SOCS3*, *RGS13*, *FDCSP*, *IGSF6*, *IRF8*, *HLA-DRB1*, *PLA2G7*, *TIMD4*, *CCL21* and *KLF10*). The primary shared functions of the differentially regulated transcripts included germinal center formation and B cell activity. SOCS3 is a negative regulator of cytokine expression through the JAK-STAT signal transduction pathway [37] and its reduced expression is consistent with a pro-inflammatory state. RGS13, which we discuss in detail below (in a separate section), regulates responsiveness of B-cells to chemokines and controls GC formation in the spleen [31, 38]. Follicular dendritic cell secreted protein (*FDCSP*), which was increased four-fold in the GC-positive group, has been identified as a regulator of B cell responsiveness to antigen stimulation [39] and GC formation [40]. Immunoglobulin super family 6 (*IGSF6*) is upregulated with antigen stimulation of dendritic cells [41, 42], and its increased expression indicates dendritic cell activation. Interferon regulatory factor 8 (*IRF-8*) is a transcriptional factor, which contributes to the early development of B cells [43] is increased in GC positive samples. IRF8 reduces signaling via the B cell antigen receptor (BCR) allowing antigen-specific interaction with helper T cells, and thereby promote antibody affinity maturation [44]. Of particular interest, is that the alpha subunit of the *ACHR* promotor region contains a functional bi-allelic variant that is associated with early onset of disease, which is the population involved in our study. IRF-8 does not bind to this variant and thereby abolishes promoter activity in thymic epithelial cells [45]. IRF-8 would then serve to modulate expression of the ACHR in thymus and thereby influence the balance supporting autoimmunity and tolerance. Increased expression of MHC class II components which has been observed by others [46], is consistent with the greater number of lymphocytes present in specimens with the GCs, and also supports elevated antigen presentation in the pathological thymus.

### Cell death and survival

The transcriptional profile supports the GC of myasthenic thymus as a site of active cell proliferation and differentiation as evidenced by the suppressed expression of the transcriptional factors FOSB, JUNB, EGR1, and EGR3 [47]. Two differentially regulated transcripts (*TIMD4*, *GADD45B*) would promote inflammation or autoimmunity through involvement of cell death and survival pathways. T-cell immunoglobulin mucin gene family members serve as regulators of multiple immune response phases and TIMD4 is involved in apoptosis of Th1 cells [48, 49]. The increased expression would be expected to further enhance a Th2-mediated inflammation. GADD45B is involved in Th1-response, and transgenic mice with a knockout of GADD45B develop a severe form of experimental autoimmune encephalomyelitis [50].

### Cell migration

Affinity maturation of B cells occurs in concert with the movement of cells through the GC [51]. Both metalloproteinase 9 (*MMP9*) and *VCAM1* were elevated in GC positive specimens. VCAM1 activates MMP9, facilitating the migration of lymphocytes across endothelial cells [52]. MMP9 acts in local extracellular matrix proteolysis and leukocyte migration [53]. An inhibitor of MMP9, TIMP2, was reduced in expression, which would further promote cell migration activity [54, 55]. The elevation of MMP9 has not been appreciated in GC but is consistent with the requirement for lymphoctyes to move through the GC environment. Expression of both *VCAM1* and *MMP9* is negatively correlated with that of *FOS* and *JUN* (S4B Fig). A characteristic finding of pathological thymus is the presence of high endothelial venules (HEV), where circulating lymphocytes gain access to secondary lymphoid organs [56]. VCAM and MMP9 are likely to be involved in processes at the HEV. Consistent with previous studies, *CCL21* was upregulated in GC rich thymus [46, 57] and likely supports GC formation in myasthenic thymus as in other secondary lymphoid organs. The study by Cizeron-Clairac et al suggests the influence of glucocorticoids in the reduction of CCL21 to normal values [34]. *ADAMDEC1*, a disintegrin metalloproteinase family member, was upregulated and part of the functional network with *MMP9* (Fig 5). ADAMADEC1 is known to support dendritic cell maturation through interaction with germinal center T cells [58]. In contrast, *ADAMTS1*, another metalloproteinase [59] and *PXN*, an adaptor protein [60], were both reduced in expression despite their actions to support cell migration. Their reduced expression suggests there is a balance of signals promoting and inhibiting cell migration in the myasthenic thymus.

### MiRNA profile of MG thymus

MicroRNA profiling distinguished GC-positive and GC-negative thymus specimens based on PCA and hierarchical clustering analyses. The miRNA profile was similar in general categorization to the transcriptional profile. IPA miRNA target filter analysis indicated an abundance of miRNAs involved in various aspects of the immune response including cytokine regulation and NF kappa B signaling as well as humoral and cellular immunity. MiRNAs identified to be involved in regulation of cancer formation and apoptosis also overlapped with the RNA profile. Cellular functions that were targets of the miRNAs included cell growth and proliferation, development and cell death/survival. The results are consistent with previous work that found similar general categories [61] and are expected given the activity of the MG thymus as the site of the generation of the autoimmune response. MG patients have reduced expression of miR320a, [17] which has been found in many cancers to be associated with enhanced tumor development. The let-7 family of miRNAs, which moderate FAS-mediated apoptosis, is also decreased in PBMCs of MG patients [62, 63]. Several miRNAs (Fig 8) likely to contribute to GC maintenance were identified [64].

Other miRNA with regulatory roles in immune systems have been identified in other studies of human MG and animal models of MG. MiR-150-5p expression was increased in sera of MG patients [23]. Circulating levels of miR-150-5p are reduced with thymectomy and correlated with disease improvement [23]. MiR-150-5p is known to influence growth, maturation, and the immune response in both B and T cells and is critical for plasma cell survival [65–67]. MiR-150 is highly expressed in naive B cells and limits the magnitude of the GC development [67] and therefore appears to be increased as a regulator and not necessarily as a driver of inflammation. Upregulation of miR-24-3p was appreciated as a direct mediator of human plasma cell survival [68]. MiR-24-3p is expressed in B cell precursors, dysregulated T cells, and malignant cells [68–71].

MicroRNAs that are involved in T cell functions were identified. MiR-142-3p and 5p expression is increased in GC positive group. These are T-cell specific miRNA [72] and regulate T cell development [73, 74]. MicroRNAs involved in NF-kappa B pathways have been identified. NF-kappa B signaling pathway is important for MG pathophysiology [1]. MiR-342-3p expression was increased in GC positive samples and has been reported to down regulate NF-kB activity pathway by targeting *IKBKG*, *TAB2* and *TAB3* genes [75]. In contrast, miR-342-3p expression is decreased in SLE patients with active nephritis [76]. MiR-145, miR-24, and miR143 were all found to be reduced in PBMC of EAMG rats [66] as was the case in our profiling. MiR-145 promotes pathogenic T cell responses, while miR-24 modulates T cell effector responses [77]. In contrast, we did not see the same alterations miR-15, miR122, miR-140-3p, miR185, miR192, miR20b and miR-885-5p as others who investigated circulating miRNA [78, 79]. This discrepancy likely can be attributed to thymus specific miRNA expression.

### MiRNA profile and autoimmune disorders

Several miRNAs were identified, which are known to be involved in other autoimmune disorders, in particular systemic lupus erythematosus, autoimmune thyroid disease, and rheumatoid arthritis, but only two were shared with multiple sclerosis. Each of the three disorders have higher than expected frequencies of co-occurrence with MG [80–84] and is part of a more general similarity of genetic risk for development of organ-specific and systemic autoimmune disorders, which is not shared with multiple sclerosis [85, 86]. In the blood, miRNA 22-3p, 24-3p, 378d and 4723-5p expression overlap across rheumatoid arthritis, systemic lupus erythematosus, autoimmune thyroid disease and our study. Each of these miRNA impact cell cycle regulation, growth, and apoptosis. MiR-22-3p had reduced expression in the GC positive group. MiR-22-3p is involved in PTEN mediated B-cell receptor (BCR) signaling and is considered to be involved in B-cell hyperactivity autoimmune systemic lupus erythematosus [87]. MiRs 24-3p and 4723-5p has been found to enhance cell proliferation in cancers, while there is limited information on miR 4723-5p. MiR-24-3p also supports human plasma cell survival [68].

### Reciprocal pairing analysis

Despite the common application of broad-based profiling methods to evaluate gene and miRNA expression, few studies have matched miRNA and gene expression profiles derived from a set of common specimens. We found 11 matches of gene transcripts with high to moderate likelihood to be regulated by corresponding miRNAs. The reduced expression of Kruppel-Like Factor 10 (*KLF10*) in GC-positive samples is particularly noteworthy. KLF10 enhances T cell suppressor function [88], which suggests an active role in promotion of the pro-inflammatory state of the MG thymus. MiRNA-RNA target pairing analysis identified three miRNAs that have predicted target sites to regulate *KLF10* expression by TargetScan. In particular, miR142-5p is predicted to moderate *KLF10* at moderately high confidence level and in our miRNA profile was expressed at an eight-fold higher level in GC positive thymus. Assuming that miR142-5p acts primarily to reduce *KLF10* expression this would be a mechanism to limit T cell suppressor activity and thereby support reaction against self-antigens. MiR-718 was identified to have high likelihood for regulation of EGR3 and recently was confirmed to directly regulate EGR3 in hepatic carcinoma cell lines [89]. Regarding the remaining pairs, there have been no experimental reports to confirm the regulation of the given miRNA on gene target regulation.

### *RGS13* expression and miRNA expression moderation

Reciprocal pairing identified miR-452-5p and miR-139-5p as potential regulators of *RGS13*. Because of its known influence on germinal center maintenance in secondary lymphoid organs, we proceeded to confirm the regulatory potential of these miRNAs. The 3’UTR of RGS13 has binding sites for these miRNAs as validated by dual luciferase assay. We found that miR-139-5p and miR-452-5p independently suppressed RGS13 expression in a lymphocytic cell line. RGS13 is thought to regulate expansion and differentiation of B cells as well as modulation of GC size [31, 38]. BXD2 mice that have spontaneously developing systemic autoimmunity with enlarged GC and autoantibody production have elevated RGS13 levels, and *RGS13* gene knockout leads to moderation of antibody production [90]. These observation suggests that mimics of miR-139-5p and miR-452-5p could be utilized as a therapeutic strategy for patients with early-onset AChR antibody positive MG.

## Conclusions

Our results suggest that development and maintenance of germinal centers involves cellular processes that are common to neoplastic pathway such as cell proliferation, reorganization of cellular matrix, cell migration and apoptosis as evidenced by profiles of gene transcription and miRNA. Gene transcripts that are responsible for cellular growth and proliferation *FOS-JUN*, *EGR1*, and *EGR3* were downregulated in the mRNA profile. For organization of GC and B cell maturation, cellular movement and cell matrix reorganization is required and such pathways were identified. The GC-rich thymus was found to have a transcriptional profile consistent with state of inflammation. The results begin to provide a comprehensive picture of the pathological thymus and identify key target genes and miRNA that could be targeted for therapeutic development.

## Supporting information

S1 Appendix Names of all local review boards and ethics committees of the participating sites. (DOCX)

S1 Fig Gradation of thymus specimens. Thymus blocks were assessed for the presence of germinal centers (GC) by hematoxylin and eosin staining. A and B are representative sections of GC positive samples; C and D are GC negative samples. Images were captured using a Leica DM 600-B microscope (Leica Microsystems Inc., Buffalo Grove, IL) at 50X magnification.
(TIF)

S2 Fig Quantitative RT-PCR validation of differentially regulated miRNA in thymus samples with and without GCs. Each dot represents data obtained from a patient. It is expressed as +/- SEM. The level of expression was normalized to that of the small noncoding snoU6 RNA. Student’s t-test was performed on relative expression level, p<0.05 is considered as significant.
(TIF)

S3 Fig Quantitative RT-PCR validation of differentially regulated mRNA in thymus samples with and without GCs. Each dot represents data obtained from a patient. It is expressed as +/- SEM. The level of expression was normalized to house-keeping gene *EIF1AX*. Student’s t-test was performed on relative expression level, p<0.05 is considered as significant.
(TIF)

S4 Fig IPA core analysis program identified network functions for the differentially expressed genes. (A) Neurological disease, organismal injury and abnormalities, hematological system development and function network are shown. (B) Cell death and survival, cellular development, cellular growth and proliferation network are depicted. The solid arrows indicate direct interactions and the dotted arrows indicate indirect interaction between the differentially expressed mRNA. The mRNAs that are over expressed in GC positive samples are marked in red and those repressed are marked in green.
(TIF)

S5 Fig *RGS13* expression in Raji cell line. *RGS13* expression was validated by RT-PCR in Raji cell line. *GAPDH* (human) was used as a control.
(TIF)

S1 Table Primer sequences. (A) Primer sequences for qRT-PCR validation of miRNA. (B) Primer sequences for qRT-PCR validation of mRNA.
(DOCX)

S2 Table (A) Differentially expressed small non coding RNA in GC positive vs GC negative thymus samples with greater than 1.5 fold change in expression (ANOVA<0.05). (B) Thirty eight matured miRNA with greater than 1.5 fold change in expression between the two groups and validation of selected miRNA expression by qRT-PCR. qRT-PCR data was normalized to the expression of snoU6 RNA. Student’s t-test was performed, p <0.05 is considered as significant (marked in bold). ND, not determined.
(DOCX)

S3 Table (A) Differentially expressed miRNAs involved in immune response pathways as identified by IPA miRNA target filter analysis (confidence level high and experimentally observed). (B) Differentially expressed miRNAs involved in cell cycle regulation and cancer pathways as identified by IPA miRNA target filter analysis (confidence level high and experimentally observed). (C) Differentially expressed miRNAs involved in autoimmune disease pathways as identified by IPA miRNA target filter analysis (confidence level high and experimentally observed).
(DOCX)

S4 Table Validation of mRNA array results by qRT-PCR. The qRT-PCR data has been normalized to the expression of housekeeping gene *EIF1AX*. Student’s t-test was performed, p<0.05 is considered as significant (in bold).
(DOCX)
