# Supplementary material for: MicroRNA and mRNA expression associated with ectopic germinal centers in thymus of myasthenia gravis
Source: PLoS One. 2018 Oct 11;13(10):e0205464. doi: 10.1371/journal.pone.0205464 (PMC6181382; doi:10.1371/journal.pone.0205464)
Supplement: S1 Appendix — (DOCX) [file pone.0205464.s001.docx]

Sites received local institutional review board/ethics committee approvals, and each patient provided written informed consent before study entry including provision of thymic specimens. All specimens were deidentified. The George Washington University institutional review board provided additional review and approved these investigations.

**Names of all local review board ethics/committees**

1. Univ. at Buffalo Jacobs School of Medicine and Biomedical Sciences, Buffalo, NY, USA
2. George Washington University School of Medicine and Health Sciences, Washington, D.C., USA
3. Univ. of Alabama at Birmingham, Birmingham, AL, USA
4. University Medical Centre Mannheim, University of Heidelberg, Mannheim, Germany
5. University of Göttingen, Göttingen, Germany
6. University of Buenos Aires, Buenos Aires, Argentina
7. University of British Columbia, Vancouver, Canada
8. University of Chile, Santiago, Chile
9. University of Cape Town, Cape Town, South Africa
10. Catholic University, Rome, Italy
11. Johanes Gutenberg University, Mainz, Germany
12. University of Rochester Medical Center, Rochester, NY, USA
13. Mental Health and Sensory Organs, University of Rome “Sapienza,” Rome, Italy
14. Mahidol University, Bangkok, Thailand
15. University of Melbourne, Melbourne, Australia
16. University of Southern California, Los Angeles, CA, USA
17. McGill University, Montreal, Canada
18. Medical College of Wisconsin, Milwaukee, WI, USA
19. Harvard Medical School, Boston, MA, USA
20. Nerve and Muscle Center of Texas, Houston, Texas, USA
21. Case Western Reserve University, Cleveland, OH, USA
22. Walton Centre for Neurology and Neurosurgery, Liverpool, U.K.
23. Oxford University, Oxford, U.K.
24. Hospital de Base do Distrito Federal, Brasília, Brazil
25. Kanazawa University, Kanazawa, Japan
26. Federal University, Rio de Janeiro, Brazil
27. University of Florida, Jacksonville, FL, USA
28. Georgia Regents University, Augusta, GA, USA
29. Medical University of Warsaw, Warsaw, Poland
30. Indiana University School of Medicine, Indianapolis, IN, USA
31. University of Texas Health Science Center, San Antonio, TX, USA
32. Leiden University Medical Center, Leiden, The Netherlands
33. Duke University Medical Center, Durham, NC, USA
34. The Ohio State University Wexner Medical Center, Columbus, OH, USA
35. Universidade Federal do Parana, Curitiba, Brazil
36. University of Miami, Miami, FL, USA
37. University of Kansas Medical Center, Kansas City, KS, USA
38. University of Vermont College of Medicine, Burlington, VT, USA
39. University of California, Irvine, Orange, CA, USA
40. NIH/National Institute of Neurological Disorders and Stroke, Bethesda, MD, USA
41. Columbia University Medical Center, New York, NY, USA:
