# Supplementary figures and images for: MicroRNA and mRNA expression associated with ectopic germinal centers in thymus of myasthenia gravis
Source: PLoS One. 2018 Oct 11;13(10):e0205464. doi: 10.1371/journal.pone.0205464 (PMC6181382; doi:10.1371/journal.pone.0205464)

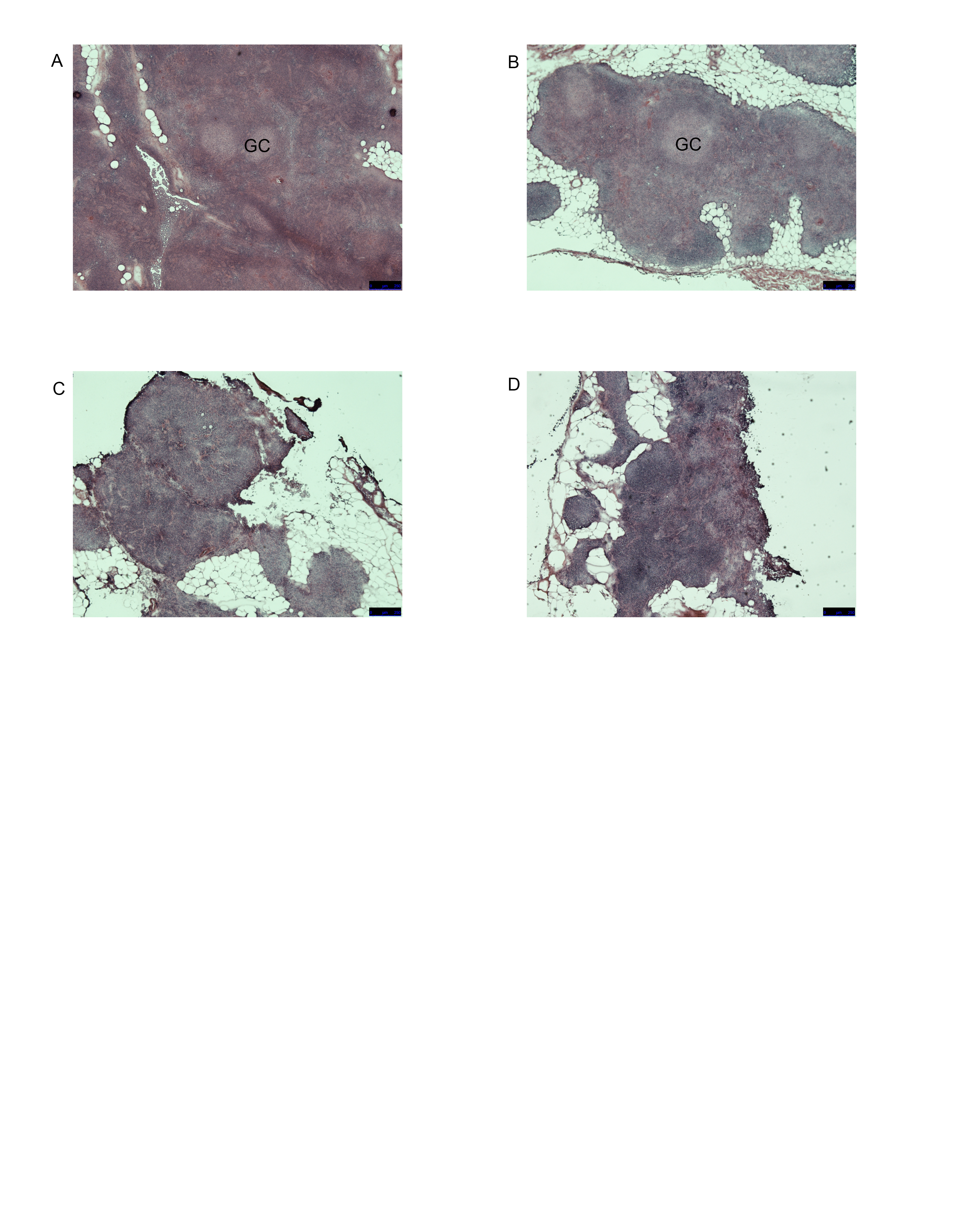

Supplement: S1 Fig — Thymus blocks were assessed for the presence of germinal centers (GC) by hematoxylin and eosin staining. A and B are representative sections of GC positive samples; C and D are GC negative samples. Images were captured using a Leica DM 600-B microscope (Leica Microsystems Inc., Buffalo Grove, IL) at 50X magnification. (TIF) [file pone.0205464.s002.tif]

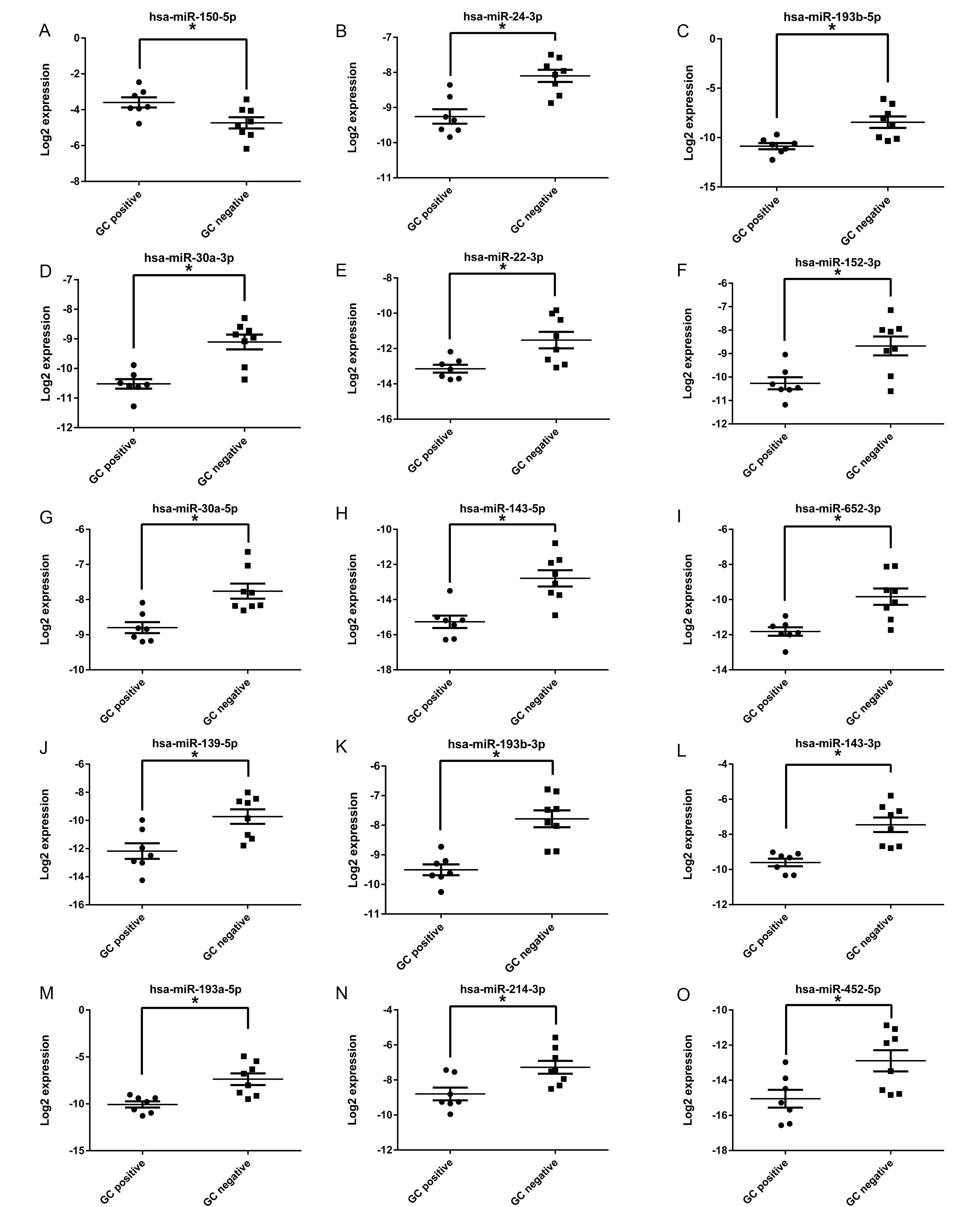

Supplement: S2 Fig — Each dot represents data obtained from a patient. It is expressed as +/- SEM. The level of expression was normalized to that of the small noncoding snoU6 RNA. Student’s t-test was performed on relative expression level, p<0.05 is considered as significant. (TIF) [file pone.0205464.s003.tif]

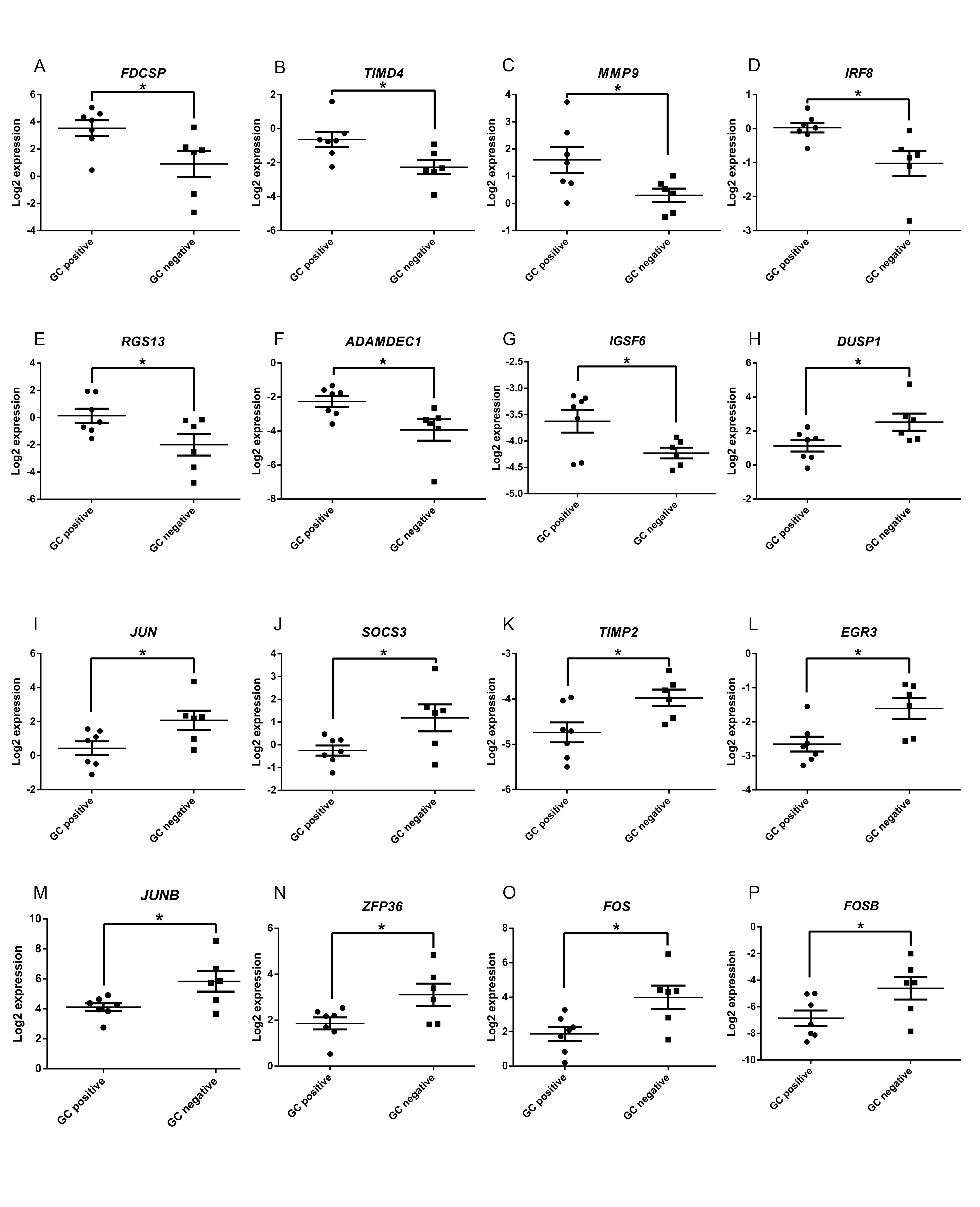

Supplement: S3 Fig — Each dot represents data obtained from a patient. It is expressed as +/- SEM. The level of expression was normalized to house-keeping gene EIF1AX. Student’s t-test was performed on relative expression level, p<0.05 is considered as significant. (TIF) [file pone.0205464.s004.tif]

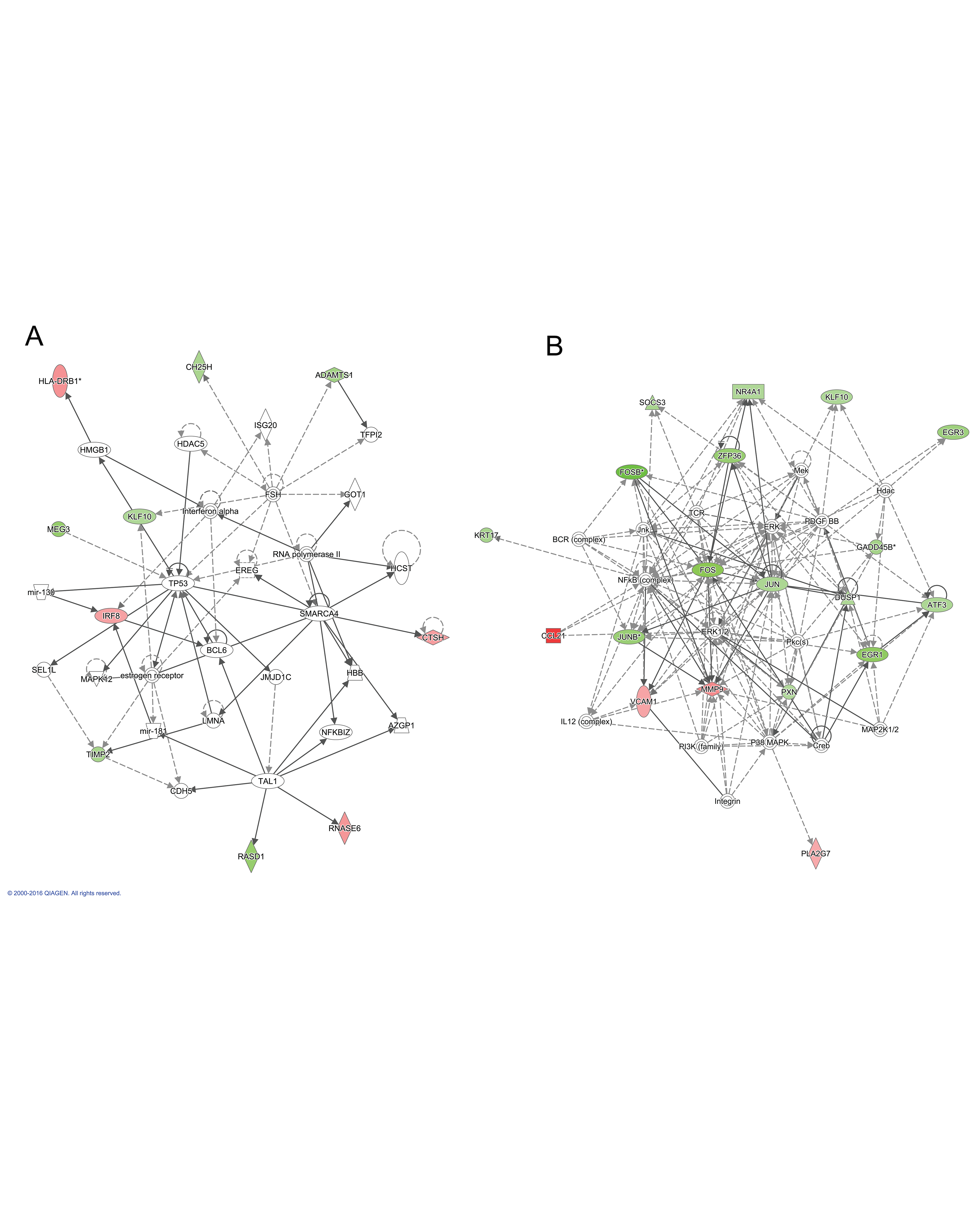

Supplement: S4 Fig — (A) Neurological disease, organismal injury and abnormalities, hematological system development and function network are shown. (B) Cell death and survival, cellular development, cellular growth and proliferation network are depicted. The solid arrows indicate direct interactions and the dotted arrows indicate indirect interaction between the differentially expressed mRNA. The mRNAs that are over expressed in GC positive samples are marked in red and those repressed are marked in green. (TIF) [file pone.0205464.s005.tif]

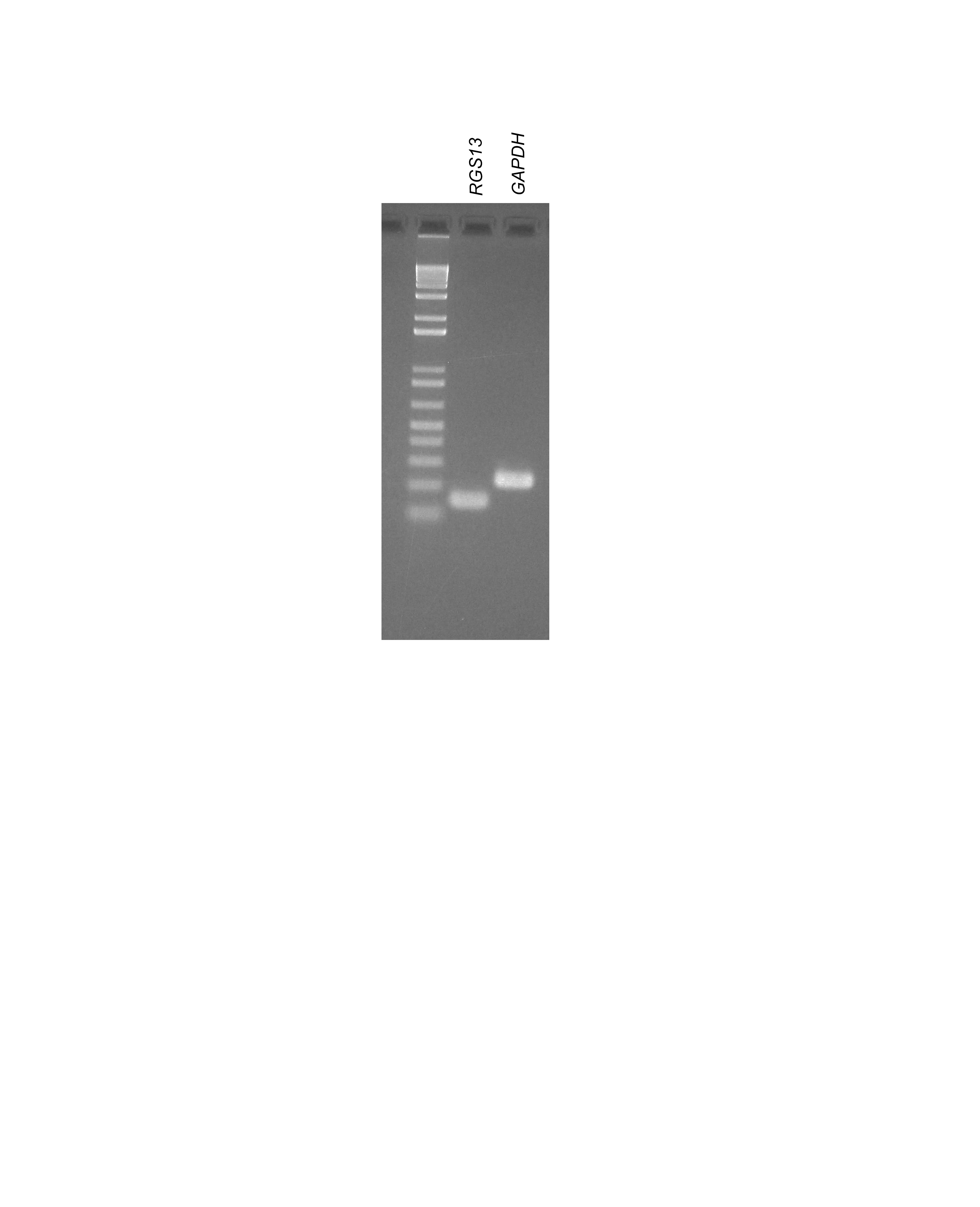

Supplement: S5 Fig — RGS13 expression was validated by RT-PCR in Raji cell line. GAPDH (human) was used as a control. (TIF) [file pone.0205464.s006.tif]
