# Supplementary material for: MicroRNA and mRNA expression associated with ectopic germinal centers in thymus of myasthenia gravis
Source: PLoS One. 2018 Oct 11;13(10):e0205464. doi: 10.1371/journal.pone.0205464 (PMC6181382; doi:10.1371/journal.pone.0205464)
Supplement: S1 Table — (A) Primer sequences for qRT-PCR validation of miRNA. (B) Primer sequences for qRT-PCR validation of mRNA. (DOCX) [file pone.0205464.s007.docx]

S1A Table: Primer sequences for qRT-PCR validation of miRNA

| **miRNA** | **Forward Primer sequence 5' - 3'** | | | |
| --- | --- | --- | --- | --- |
|  |  |  |  |  |
| hsa-miR-486-5p | TCCTGTACTGAGCTGCCCCGAG | | | |
| hsa-miR-452-5p | AACTGTTTGCAGAGGAAACTGA | | | |
| hsa-miR-378d | ACTGGACTTGGAGTCAGAAA | | |  |
| hsa-miR-214-3p | ACAGCAGGCACAGACAGGCAGT | | | |
| hsa-miR-193a-5p | TGGGTCTTTGCGGGCGAGATGA | | | |
| hsa-miR-143-5p | GGTGCAGTGCTGCATCTCTGGT | | | |
| hsa-miR-574-3p | CACGCTCATGCACACACCCACA | | | |
| hsa-miR-143-3p | TGAGATGAAGCACTGTAGCTC | | |  |
| hsa-miR-193b-3p | AACTGGCCCTCAAAGTCCCGCT | | | |
| hsa-miR-139-5p | TCTACAGTGCACGTGTCTCCAGT | | | |
| hsa-miR-652-3p | AATGGCGCCACTAGGGTTGTG | | |  |
| hsa-miR-145-5p | GTCCAGTTTTCCCAGGAATCCCT | | | |
| hsa-miR-30a-5p | TGTAAACATCCTCGACTGGAAG | | | |
| hsa-miR-22-3p | AAGCTGCCAGTTGAAGAACTGT | | | |
| hsa-miR-152-3p | TCAGTGCATGACAGAACTTGG | | |  |
| hsa-miR-198 | GGTCCAGAGGGGAGATAGGTTC | | | |
| hsa-miR-30a-3p | CTTTCAGTCGGATGTTTGCAGC | | | |
| hsa-miR-193b-5p | CGGGGTTTTGAGGGCGAGATGA | | | |
| hsa-miR-24-3p | TGGCTCAGTTCAGCAGGAACAG | | | |
| hsa-miR-150-5p | TCTCCCAACCCTTGTACCAGTG | | | |
| hsa-miR-718 | CTTCCGCCCCGCCGGGCGTCG | | | |
| hsa-miR-342-3p | TCTCACACAGAAATCGCACCCGT | | | |
| hsa-miR-142-3p | TGTAGTGTTTCCTACTTTATGGA | | | |
| hsa-miR-142-5p | CATAAAGTAGAAAGCACTACT | | |  |
| U6snRNA | CGCAAGGATGACACGCAA | | |  |
|  |  |  |  |  |

S1B Table: Primer sequences for qRT-PCR validation of mRNA

| **Gene Symbol** | **RefSeq** | **Forward Primer Sequence 5' to 3'** | **Reverse Primer Sequence 5' to 3'** | | |
| --- | --- | --- | --- | --- | --- |
|  |  |  |  |  |  |
| *FDCSP* | NM_152997 | TCTCTCAAGACCAGGAACGA | GGAAATGGAATTGGTGGAAG | | |
| *CCL21* | NM_002989 | CTCCATCCCAGCTATCCTGT | TGGTGTCTTGTCCAGATGCT | | |
| *TIMD4* | NM_001146726 | CATTGTTTGTGGCGTTTCTC | GCACGTCATTGAGGACATTT | | |
| *MMP9* | NM_004994 | TCCAGTACCGAGAGAAAGCC | GCAGGATGTCATAGGTCACG | | |
| *HLA-DRB1* | NM_001243965 | CGGGCTGTTCATCTACTTCA | ATCTGCACTTCAGCTCAGGA | | |
| *IRF8* | NM_002163 | ATGAGGTGGTCCAGGTCTTC | GCCATATCCGGAAACTCTTC | | |
| *RGS13* | NM_002927 | TTTACATCCAGCCACAGTCC | GGGTAGGAATCCCTTTCCAT | | |
| *ADAMDEC1* | NM_001145271 | GACTGTGATTGTGGCTCTCC | TCCAGGCTTCAGTTTACACG | | |
| *IGSF6* | NM_005849 | CGGAAGCGAGAGCTAAACA | AGTTCCTTGCTGAGCAGCTT | | |
| *PXN* | BC052611 | GCCTAGGCAACACAGTGAAA | AAAGCCATCCTCCTGCTTTA | | |
| *DUSP1* | NM_004417 | CCCTGAGTACTAGCGTCCCT | ACTGCCCAGGTACAGAAAGG | | |
| *ATF3* | NM_001030287 | GCCGAAACAAGAAGAAGGAG | GCTCCTCAATCTGAGCCTTC | | |
| *GADD45B* | AY615271 | CTGCATTGTCTCCTGGTCAC | TCAGCGTTCCTGAAGAGAGA | | |
| *JUN* | NM_002228 | CAGCCCACTGAGAAGTCAAA | CACCAATTCCTGCTTTGAGA | | |
| *SOCS3* | NM_003955 | CTTCCTGTACCTGGGTGGAT | GGCGAATCTCTTAGCCAGAC | | |
| *TIMP2* | X54533 | CCCAGAAGAAGAGCCTGAAC | GAGGAGATGTAGCACGGGAT | | |
| *ADAMTS1* | NM_006988 | TGATGGTTTACAAGCTGCCT | ACCATTAAGGCTGGCACACT | | |
| *EGR3* | S40832 | GCTGAACTGGGCTGTGTTTA | ACATGATTTCAGAGCGGATG | | |
| *JUNB* | NM_002229 | CCATCAACATGGAAGACCAA | TTGAGCGTCTTCACCTTGTC | | |
| *ZFP36* | NM_003407 | TTTAAGGGAGGCAATGAACC | CAGGAGACACTGGAACCTCA | | |
| *RASD1* | NM_001199989 | GCCTCTCCATCCTCACAGAT | GTCCACGTTCTCCTTGGTTT | | |
| *EGR1* | NM_001964 | CAGCACCTTCAACCCTCAG | GCACCTTCTCGTTGTTCAGA | | |
| *FOS* | NM_005252 | TACACTCCAAGCGGAGACAG | TCCTTCTCCTTCAGCAGGTT | | |
| *FOSB* | NM_001114171 | CTACTCCACACCAGGCATGA | TGATCTGTCTCCGTCTCCTCT | | |
| EIF1AX | NM_001412.3 | GTACTGGAGAGGGGAGAGCA | TGAAGCTGAGACAAGCAGGA | | |
| hGAPDH | KJ891221.1 | GAAGGTGAAGGTCGGAGTC | GAAGATGGTGATGGGATTTC | | |
