# Supplementary material for: MicroRNA and mRNA expression associated with ectopic germinal centers in thymus of myasthenia gravis
Source: PLoS One. 2018 Oct 11;13(10):e0205464. doi: 10.1371/journal.pone.0205464 (PMC6181382; doi:10.1371/journal.pone.0205464)
Supplement: S2 Table — (A) Differentially expressed small non coding RNA in GC positive vs GC negative thymus samples with greater than 1.5 fold change in expression (ANOVA<0.05). (B) Thirty eight matured miRNA with greater than 1.5 fold change in expression between the two groups and validation of selected miRNA expression by qRT-PCR. qRT-PCR data was normalized to the expression of snoU6 RNA. Student’s t-test was performed, p <0.05 is considered as significant (marked in bold). ND, not determined. (DOCX) [file pone.0205464.s008.docx]

S2A Table: Differentially expressed small non coding RNA in GC positive vs GC negative thymus samples with greater than 1.5 fold change in expression (ANOVA<0.05)

| **Transcript Cluster ID** | **Transcript ID** | **Accession** | **Fold Change** | **ANOVA p-value** |  |
| --- | --- | --- | --- | --- | --- |
|  |  |  |  |  |  |
| 20500749 | hsa-miR-142-5p | MIMAT0000433 | 8.02 | 0.0336 |  |
| 20500750 | hsa-miR-142-3p | MIMAT0000434 | 5.05 | 0.0421 |  |
| 20513597 | hsa-miR-718 | MIMAT0012735 | 2.48 | 0.0200 |  |
| 20534179 | ENSG00000266284 | ENSG00000266284 | 2.36 | 0.0452 |  |
| 20538168 | U36A | U36A | 2.36 | 0.0452 |  |
| 20538203 | U54 | U54 | 2.2 | 0.0166 |  |
| 20501280 | hsa-miR-342-3p | MIMAT0000753 | 2.04 | 0.0384 |  |
| 20534306 | HBII-85-14 | HBII-85-14 | 1.92 | 0.0142 |  |
| 20534335 | HBII-95 | HBII-95 | 1.77 | 0.0484 |  |
| 20538163 | U32B | U32B | 1.68 | 0.0340 |  |
| 20535324 | hsa-mir-584 | MI0003591 | 1.66 | 0.0049 |  |
| 20536638 | hsa-mir-3928 | MI0016438 | 1.65 | 0.0025 |  |
| 20536686 | hsa-mir-4436a | MI0016776 | 1.62 | 0.0042 |  |
| 20538304 | snR39B | snR39B | 1.6 | 0.0097 |  |
| 20537018 | hsa-mir-4773-1 | MI0017415 | 1.59 | 0.0142 |  |
| 20500782 | hsa-miR-150-5p | MIMAT0000451 | 1.58 | 0.0116 |  |
| 20504296 | hsa-miR-573 | MIMAT0003238 | 1.58 | 0.0238 |  |
| 20504352 | hsa-miR-611 | MIMAT0003279 | 1.53 | 0.0028 |  |
| 20532870 | ENSG00000201541 | ENSG00000201541 | 1.52 | 0.0473 |  |
| 20534326 | HBII-85-3 | HBII-85-3 | 1.52 | 0.0311 |  |
| 20534332 | HBII-85-9 | HBII-85-9 | 1.52 | 0.0311 |  |
| 20515636 | hsa-miR-3194-3p | MIMAT0019218 | 1.51 | 0.0224 |  |
| 20534322 | HBII-85-27 | HBII-85-27 | 1.51 | 0.0053 |  |
| 20537623 | hsa-mir-6877 | MI0022724 | -1.51 | 0.0208 |  |
| 20500189 | hsa-miR-29b-2-5p | MIMAT0004515 | -1.53 | 0.0426 |  |
| 20536315 | hsa-mir-3118-5 | MI0014243 | -1.56 | 0.0196 |  |
| 20509231 | hsa-miR-1911-3p | MIMAT0007886 | -1.59 | 0.0418 |  |
| 20500433 | hsa-miR-139-3p | MIMAT0004552 | -1.6 | 0.0203 |  |
| 20500148 | hsa-miR-24-3p | MIMAT0000080 | -1.62 | 0.0008 |  |
| 20526861 | hsa-miR-7150 | MIMAT0028211 | -1.68 | 0.0267 |  |
| 20519559 | hsa-miR-4723-5p | MIMAT0019838 | -1.77 | 0.0325 |  |
| 20503807 | hsa-miR-193b-5p | MIMAT0004767 | -1.78 | 0.0076 |  |
| 20500163 | hsa-miR-30a-3p | MIMAT0000088 | -1.79 | 0.0259 |  |
| 20500396 | hsa-miR-198 | MIMAT0000228 | -1.85 | 0.0235 |  |
| 20525535 | hsa-miR-6787-5p | MIMAT0027474 | -1.88 | 0.0397 |  |
| 20500144 | hsa-miR-22-3p | MIMAT0000077 | -1.89 | 0.0221 |  |
| 20500758 | hsa-miR-152-3p | MIMAT0000438 | -1.91 | 0.0045 |  |
| 20500162 | hsa-miR-30a-5p | MIMAT0000087 | -1.94 | 0.0024 |  |
| 20524036 | hsa-miR-6126 | MIMAT0024599 | -1.98 | 0.0380 |  |
| 20500799 | hsa-miR-195-3p | MIMAT0004615 | -1.99 | 0.0152 |  |
| 20500751 | hsa-miR-143-5p | MIMAT0004599 | -2.01 | 0.0035 |  |
| 20500755 | hsa-miR-145-5p | MIMAT0000437 | -2.07 | 0.0092 |  |
| 20504408 | hsa-miR-652-3p | MIMAT0003322 | -2.08 | 0.0139 |  |
| 20500432 | hsa-miR-139-5p | MIMAT0000250 | -2.09 | 0.0331 |  |
| 20503808 | hsa-miR-193b-3p | MIMAT0002819 | -2.11 | 0.0195 |  |
| 20500752 | hsa-miR-143-3p | MIMAT0000435 | -2.12 | 0.0018 |  |
| 20518783 | hsa-miR-378e | MIMAT0018927 | -2.14 | 0.0353 |  |
| 20504298 | hsa-miR-574-3p | MIMAT0003239 | -2.15 | 0.0177 |  |
| 20501176 | hsa-miR-99b-5p | MIMAT0000689 | -2.3 | 0.0250 |  |
| 20500795 | hsa-miR-193a-5p | MIMAT0004614 | -3.02 | 0.0120 |  |
| 20500472 | hsa-miR-214-3p | MIMAT0000271 | -3.32 | 0.0360 |  |
| 20502122 | hsa-miR-422a | MIMAT0001339 | -3.41 | 0.0469 |  |
| 20504584 | hsa-miR-378d | MIMAT0018926 | -3.5 | 0.0478 |  |
| 20502451 | hsa-miR-452-5p | MIMAT0001635 | -3.99 | 0.0113 |  |
| 20503105 | hsa-miR-486-5p | MIMAT0002177 | -6.36 | 0.0410 |  |
|  |  |  |  |  |  |
|  |  |  |  |  |  |

S2B Table: Thirty eight matured miRNA with greater than 1.5 fold change in expression between the two groups and validation of selected miRNA expression by qRT-PCR. qRT-PCR data was normalized to the expression of snoU6 RNA. Student t-test was performed, p <0.05 is considered as significant (marked in bold). ND, not determined.

| **Mature miRNA Transcript ID** | **Accession** | **Array Fold Change** | **Array ANOVA p-value** | **qRT-PCR Relative expression** | **qRT-PCR T-test, p value** |
| --- | --- | --- | --- | --- | --- |
|  |  |  |  |  |  |
| hsa-miR-142-5p | MIMAT0000433 | 8.02 | 0.034 | 2.290 | 0.293 |
| hsa-miR-142-3p | MIMAT0000434 | 5.05 | 0.042 | 2.415 | 0.311 |
| hsa-miR-718 | MIMAT0012735 | 2.48 | 0.020 | 0.725 | 0.260 |
| hsa-miR-342-3p | MIMAT0000753 | 2.04 | 0.038 | 1.534 | 0.124 |
| hsa-miR-150-5p | MIMAT0000451 | 1.58 | 0.012 | 2.196 | **0.028** |
| hsa-miR-573 | MIMAT0003238 | 1.58 | 0.024 | ND | - |
| hsa-miR-611 | MIMAT0003279 | 1.53 | 0.003 | ND | - |
| hsa-miR-3194-3p | MIMAT0019218 | 1.51 | 0.022 | ND | - |
| hsa-miR-29b-2-5p | MIMAT0004515 | -1.53 | 0.043 | ND | - |
| hsa-miR-1911-3p | MIMAT0007886 | -1.59 | 0.042 | ND | - |
| hsa-miR-139-3p | MIMAT0004552 | -1.6 | 0.020 | ND | - |
| hsa-miR-24-3p | MIMAT0000080 | -1.62 | 0.001 | 0.449 | **0.002** |
| hsa-miR-7150 | MIMAT0028211 | -1.68 | 0.027 | ND | - |
| hsa-miR-4723-5p | MIMAT0019838 | -1.77 | 0.032 | ND | - |
| hsa-miR-193b-5p | MIMAT0004767 | -1.78 | 0.008 | 0.186 | **0.047** |
| hsa-miR-30a-3p | MIMAT0000088 | -1.79 | 0.026 | 0.375 | **0.001** |
| hsa-miR-198 | MIMAT0000228 | -1.85 | 0.024 | 0.156 | 0.109 |
| hsa-miR-6787-5p | MIMAT0027474 | -1.88 | 0.040 | ND | - |
| hsa-miR-22-3p | MIMAT0000077 | -1.89 | 0.022 | 0.326 | **0.034** |
| hsa-miR-152-3p | MIMAT0000438 | -1.91 | 0.005 | 0.332 | **0.017** |
| hsa-miR-30a-5p | MIMAT0000087 | -1.94 | 0.002 | 0.487 | **0.016** |
| hsa-miR-6126 | MIMAT0024599 | -1.98 | 0.038 | ND | - |
| hsa-miR-195-3p | MIMAT0004615 | -1.99 | 0.015 | ND | - |
| hsa-miR-143-5p | MIMAT0004599 | -2.01 | 0.003 | 0.180 | **0.027** |
| hsa-miR-145-5p | MIMAT0000437 | -2.07 | 0.009 | 0.151 | 0.068 |
| hsa-miR-652-3p | MIMAT0003322 | -2.08 | 0.014 | 0.253 | **0.030** |
| hsa-miR-139-5p | MIMAT0000250 | -2.09 | 0.033 | 0.184 | **0.020** |
| hsa-miR-193b-3p | MIMAT0002819 | -2.11 | 0.019 | 0.303 | **0.003** |
| hsa-miR-143-3p | MIMAT0000435 | -2.12 | 0.002 | 0.226 | **0.013** |
| hsa-miR-378e | MIMAT0018927 | -2.14 | 0.035 | ND | - |
| hsa-miR-574-3p | MIMAT0003239 | -2.15 | 0.018 | 0.262 | 0.140 |
| hsa-miR-99b-5p | MIMAT0000689 | -2.3 | 0.025 | ND | - |
| hsa-miR-193a-5p | MIMAT0004614 | -3.02 | 0.012 | 0.155 | **0.044** |
| hsa-miR-214-3p | MIMAT0000271 | -3.32 | 0.036 | 0.347 | **0.050** |
| hsa-miR-422a | MIMAT0001339 | -3.41 | 0.047 | ND | - |
| hsa-miR-378d | MIMAT0018926 | -3.5 | 0.048 | 0.283 | 0.052 |
| hsa-miR-452-5p | MIMAT0001635 | -3.99 | 0.011 | 0.224 | **0.040** |
| hsa-miR-486-5p | MIMAT0002177 | -6.36 | 0.041 | 0.189 | 0.224 |
|  |  |  |  |  |  |
|  |  |  |  |  |  |
