# Supplementary material for: MicroRNA and mRNA expression associated with ectopic germinal centers in thymus of myasthenia gravis
Source: PLoS One. 2018 Oct 11;13(10):e0205464. doi: 10.1371/journal.pone.0205464 (PMC6181382; doi:10.1371/journal.pone.0205464)
Supplement: S3 Table — (A) Differentially expressed miRNAs involved in immune response pathways as identified by IPA miRNA target filter analysis (confidence level high and experimentally observed). (B) Differentially expressed miRNAs involved in cell cycle regulation and cancer pathways as identified by IPA miRNA target filter analysis (confidence level high and experimentally observed). (C) Differentially expressed miRNAs involved in autoimmune disease pathways as identified by IPA miRNA target filter analysis (confidence level high and experimentally observed). (DOCX) [file pone.0205464.s009.docx]

S3A Table: Differentially expressed miRNAs involved in immune response pathways as identified by IPA miRNA target filter analysis (confidence level high and experimentally observed)

| **Humoral Immune response** | **Cellular Immune response** | **Cytokine signaling** | **NF kappa B** |
| --- | --- | --- | --- |
|  |  |  |  |
| 99b-5p | 99b-5p | 99b-5p | 99b-5p |
| 139-5p | 139-5p | 139-5p | 139-5p |
| 142-3p | 142-3p | 142-3p | 142-3p |
| 143-3p | - | - | - |
| - | 142-5p | 142-5p | - |
| - | 143-3p | 143-3p | 143-3p |
| 145-5p | 145-5p | 145-5p | 145-5p |
| 152-3p | 152-3p | 152-3p | 152-3p |
| 150-5p | 150-5p | 150-5p | 150-5p |
| 6787-5p | 6787-5p | 6787-5p | 6787-5p |
| 193b-3p | 193b-3p | 193b-3p | 193b-3p |
| 193a-5p | 193a-5p | 193a-5p | 193a-5p |
| 198 | 198 | 198 | 198 |
| 214-3p | 214-3p | 214-3p | 214-3p |
| 22-3p | 22-3p | 22-3p | 22-3p |
| 24-3p | 24-3p | 24-3p | - |
| 30a-3p | 30a-3p | 30a-3p | 30a-3p |
| 30a-5p | 30a-5p | 30a-5p | 30a-5p |
| 611 | 611 | 611 | - |
| 3194-3p | 3194-3p | 3194-3p | 3194-3p |
| 342-3p | 342-3p | 342-3p | 342-3p |
| 573 | 573 | 573 | - |
| 378d | 378d | 378d | 378d |
| 452-5p | 452-5p | 452-5p | - |
| 4723-5p | 4723-5p | 4723-5p | 4723-5p |
| 486-5p | 486-5p | 486-5p | 486-5p |
| 574-3p | 574-3p | 574-3p | 574-3p |
| 652-3p | 652-3p | 652-3p | 652-3p |
| 718 | 718 | 718 | - |
|  |  |  |  |
|  |  |  |  |

S3B Table: Differentially expressed miRNAs involved in cell cycle regulation and cancer pathways as identified by IPA miRNA target filter analysis (confidence level high and experimentally observed)

| **Cell Cycle Regulation** | **Cellular growth, Proliferation and Development** | **Apoptosis** | **Cancer** |
| --- | --- | --- | --- |
| 99b-5p | 99b-5p | 99b-5p | 99b-5p |
| 139-5p | 139-5p | 139-5p | 139-5p |
| 142-3p | 142-3p | 142-3p | 142-3p |
| 142-5p | 142-5p | - | - |
| 143-3p | 143-3p | 143-3p | 143-3p |
| 145-5p | 145-5p | 145-5p | 145-5p |
| 152-3p | 152-3p | 152-3p | 152-3p |
| 150-5p | 150-5p | 150-5p | 150-5p |
| 6787-5p | 6787-5p | 6787-5p | 6787-5p |
| 193b-3p | 193b-3p | 193b-3p | 193b-3p |
| - | 193a-5p | 193a-5p | - |
| 198 | 198 | 198 | 198 |
| 214-3p | 214-3p | 214-3p | 214-3p |
| 22-3p | 22-3p | 22-3p | 22-3p |
| - | 24-3p | 24-3p | - |
| 30a-3p | - | 30a-3p | 30a-3p |
| 30a-5p | - | 30a-5p | - |
| 611 | 611 | 611 | - |
| 3194-3p | 3194-3p | 3194-3p | - |
| 342-3p | 342-3p | 342-3p | 342-3p |
| 573 | 573 | - | - |
| 378d | 378d | 378d | 378d |
| 452-5p | 452-5p | - | 452-5p |
| 4723-5p | 4723-5p | 4723-5p | - |
| 486-5p | 486-5p | 486-5p | - |
| 574-3p | 574-3p | 574-3p | 574-3p |
| 652-3p | 652-3p | 652-3p | 652-3p |
| 718 | 718 | 718 | 718 |
|  |  |  |  |
|  |  |  |  |

S3C Table: Differentially expressed miRNAs involved in autoimmune disease pathways as identified by IPA miRNA target filter analysis (confidence level high and experimentally observed)

| **Systemic Lupus Erythematosus Signaling** | **Multiple Sclerosis** | **Rheumatoid Arthritis** | **Autoimmune Thyroid** |
| --- | --- | --- | --- |
|  |  |  |  |
| 99b-5p | - | - | - |
| 139-5p | - | - | - |
| 142-3p | - | 142-3p | - |
| 143-3p | - | 143-3p | 143-3p |
| 145-5p | - | 145-5p | 145-5p |
| 152-3p | - | 152-3p | 152-3p |
| 150-5p | 150-5p | 150-5p | - |
| 6787-5p | - | 6787-5p | - |
| 193b-3p | - | 193a-5p | 193a-5p |
| 193a-5p | - | - | - |
| 214-3p | - | 214-3p | 22-3p |
| 22-3p | - | 22-3p | 24-3p |
| 24-3p | - | 24-3p | - |
| 30a-3p | - | 30a-5p | - |
| 611 | - | - | - |
| 3194-3p | - | 3194-3p | - |
| 342-3p | - | - | - |
| 378d | - | 378d | 378d |
| 4723-5p | - | 4723-5p | 4723-5p |
| 486-5p | - | - | - |
| - | 573 | - | - |
| - | - | 652-3p | - |
|  |  |  |  |
|  |  |  |  |
