# Supplementary material for: MicroRNA and mRNA expression associated with ectopic germinal centers in thymus of myasthenia gravis
Source: PLoS One. 2018 Oct 11;13(10):e0205464. doi: 10.1371/journal.pone.0205464 (PMC6181382; doi:10.1371/journal.pone.0205464)
Supplement: S4 Table — The qRT-PCR data has been normalized to the expression of housekeeping gene EIF1AX. Student’s t-test was performed, p<0.05 is considered as significant (in bold). (DOCX) [file pone.0205464.s010.docx]

S4 Table: Validation of mRNA array results by qRT-PCR. The qRT-PCR data has been normalized to the expression of housekeeping gene *EIF1AX*. Student t-test was performed, p<0.05 is considered as significant (in bold).

| **Gene Symbol** | **RefSeq** | **Array Fold-Change** | **Array ANOVA p-value** | **qRT-PCR relative expression** | **qRT-PCR T-test, p value** |
| --- | --- | --- | --- | --- | --- |
|  |  |  |  |  |  |
| *FDCSP* | NM_152997 | 3.661 | 0.015 | 6.190 | **0.035** |
| *CCL21* | NM_002989 | 3.604 | 0.036 | 5.045 | 0.051 |
| *TIMD4* | NM_001146726 | 2.376 | 0.023 | 3.086 | **0.023** |
| *MMP9* | NM_004994 | 2.071 | 0.024 | 2.465 | **0.041** |
| *HLA-DRB1* | NM_001243965 | 1.922 | 0.047 | 2.091 | 0.055 |
| *IRF8* | NM_002163 | 1.655 | 0.037 | 2.069 | **0.016** |
| *RGS13* | NM_002927 | 1.613 | 0.027 | 4.369 | **0.042** |
| *ADAMDEC1* | NM_001145271 | 1.545 | 0.021 | 3.191 | **0.031** |
| *IGSF6* | NM_005849 | 1.507 | 0.012 | 1.520 | **0.035** |
| *PXN* | BC052611 | -1.544 | 0.035 | 0.549 | 0.193 |
| *DUSP1* | NM_004417 | -1.593 | 0.023 | 0.379 | **0.036** |
| *ATF3* | NM_001030287 | -1.619 | 0.015 | 0.424 | 0.069 |
| *GADD45B* | AY615271 | -1.627 | 0.008 | 0.436 | 0.078 |
| *JUN* | NM_002228 | -1.661 | 0.022 | 0.320 | **0.034** |
| *SOCS3* | NM_003955 | -1.751 | 0.006 | 0.371 | **0.035** |
| *TIMP2* | X54533 | -1.755 | 0.018 | 0.590 | **0.025** |
| *ADAMTS1* | NM_006988 | -1.914 | 0.028 | 0.413 | 0.051 |
| *EGR3* | S40832 | -2.030 | 0.012 | 0.484 | **0.016** |
| *JUNB* | NM_002229 | -2.136 | 0.008 | 0.303 | **0.030** |
| *ZFP36* | NM_003407 | -2.182 | 0.010 | 0.421 | **0.037** |
| *RASD1* | NM_001199989 | -2.225 | 0.034 | 0.454 | 0.056 |
| *EGR1* | NM_001964 | -2.439 | 0.024 | 0.346 | 0.067 |
| *FOS* | NM_005252 | -2.597 | 0.012 | 0.230 | **0.019** |
| *FOSB* | NM_001114171 | -2.837 | 0.017 | 0.210 | **0.046** |
|  |  |  |  |  |  |
